# Burying Dogs in Ancient Cis-Baikal, Siberia: Temporal Trends and Relationships with Human Diet and Subsistence Practices

**DOI:** 10.1371/journal.pone.0063740

**Published:** 2013-05-17

**Authors:** Robert J. Losey, Sandra Garvie-Lok, Jennifer A. Leonard, M. Anne Katzenberg, Mietje Germonpré, Tatiana Nomokonova, Mikhail V. Sablin, Olga I. Goriunova, Natalia E. Berdnikova, Nikolai A. Savel’ev

**Affiliations:** 1 Department of Anthropology, University of Alberta, Edmonton, Alberta, Canada; 2 Conservation and Evolutionary Genetics Group, Estación Biológica de Doñana (Consejo Superior de Investigaciones Cientificas), Seville, Spain; 3 Department of Archaeology, University of Calgary, Calgary, Alberta, Canada; 4 Department of Palaeontology, Royal Belgian Institute of Natural Sciences, Brussels, Belgium; 5 Department of Anthropology, University of British Columbia, Vancouver, BC, Canada; 6 Zoological Institute, Russian Academy of Science, Saint-Petersburg, Russia; 7 Department of Archaeology and Ethnography, Irkutsk State University, Irkutsk, Russia; 8 Department of Archaeology and Ethnography, Irkutsk State University, Irkutsk, Russia; 9 Department of Archaeology and Ethnography, Irkutsk State University, Irkutsk, Russia; New York State Museum, United States of America

## Abstract

The first objective of this study is to examine temporal patterns in ancient dog burials in the Lake Baikal region of Eastern Siberia. The second objective is to determine if the practice of dog burial here can be correlated with patterns in human subsistence practices, in particular a reliance on terrestrial mammals. Direct radiocarbon dating of a suite of the region’s dog remains indicates that these animals were given burial only during periods in which human burials were common. Dog burials of any kind were most common during the Early Neolithic (∼7–8000 B.P.), and rare during all other time periods. Further, only foraging groups seem to have buried canids in this region, as pastoralist habitation sites and cemeteries generally lack dog interments, with the exception of sacrificed animals. Stable carbon and nitrogen isotope data indicate that dogs were only buried where and when human diets were relatively rich in aquatic foods, which here most likely included river and lake fish and Baikal seal (*Phoca sibirica*). Generally, human and dog diets appear to have been similar across the study subregions, and this is important for interpreting their radiocarbon dates, and comparing them to those obtained on the region’s human remains, both of which likely carry a freshwater old carbon bias. Slight offsets were observed in the isotope values of dogs and humans in our samples, particularly where both have diets rich in aquatic fauna. This may result from dietary differences between people and their dogs, perhaps due to consuming fish of different sizes, or even different tissues from the same aquatic fauna. This paper also provides a first glimpse of the DNA of ancient canids in Northeast Asia.

## Introduction

**Figure 1:**
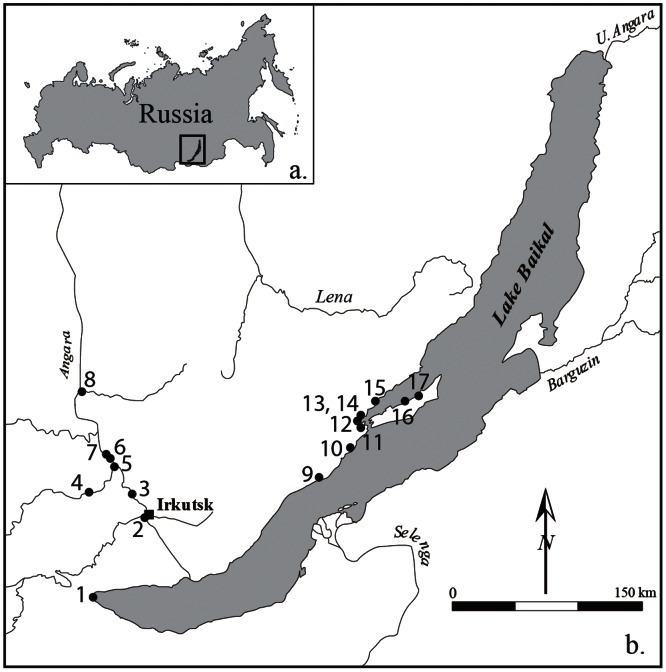
Map of the study area. Figure 1a. Location of study area within the Russian Federation. Figure 1b. Site locations mentioned in the text. Numbers 1–8 are in the Angara River/South Baikal region, and numbers 9–17 are in Priol’khon’e: 1. Shamanka II, 2. Lokomotiv, 3. Kitoi, 4. Ust’-Khaita, 5. Ust’-Belaia, 6. Pad Lenkovka, 7. Pad Kalashnikova, 8. Ust’-Ida, 9. Bugul’deika II, 10. Khotoruk, 11. Ulan-Khada, 12. Uliarba II, 13. Sarminskii Mys, 14. Khuzhir-Nuge XIV, 15. Kurma XI, 16. Shamanskii Mys, 17. Todakta I.

During the Late Pleistocene, people in Eurasia began ritually burying domesticated dogs, indicating that humans’ close personal interactions with these animals have a very deep, and likely a very complex, history [1]. Skeletal remains of ancient dogs from around the globe are increasingly being studied to better understand these animals’ evolution, life histories, and relationships with humans. Cis-Baikal, the region encompassing the southern and western shores of Eastern Siberia’s Lake Baikal and the lands surrounding the upper portions of the Angara and Lena rivers west of this lake (Figure 1), has rich collections of archaeological canid remains, including dog and wolf skeletons from graves and disarticulated remains from habitation sites [2]. This region also has one of North Asia’s most thoroughly studied Holocene archaeological records, which provides a firm culture history framework within which human-dog interaction can be explored [3].

In a previous examination of canid remains from the region, we proposed that Cis-Baikal foragers were burying select dogs and wolves because these animals had attained near-human status, and because the culturally appropriate way to care for such individuals at death that emerged during the Middle Holocene was interment in cemeteries [2]. At the time only two of the region’s canids had been directly dated, both to the Middle Holocene, and it was unknown if dogs only were buried by the foragers living here during this period, or if they were interred throughout the Holocene, including by pastoralists, who first arrived ∼3400 B.P (all dates are in calibrated years before present). Further, no detailed studies had been made on the diets of ancient dogs in Cis-Baikal and how these might have varied temporally, geographically, and in relation to human dietary and subsistence patterns. As a result, it was unknown if the practice of burying dogs could be related to the particular ways in which dogs were utilized by people in the past. For example, dog burials potentially could be most common among foraging groups that were heavily dependent on terrestrial game like deer, which can be effectively hunted with dogs. Correspondingly, dog interment then might be uncommon among foraging groups relying mostly on fishing, which likely would not have directly involved the use of dogs. Alternatively, dog burials might be widespread among pastoralists who became emotionally tied to certain dogs through their use in herding, livestock protection, and hunting. Long-term practices of dog burial in relation to such factors rarely have been directly examined with archaeological data.

This paper provides the first in-depth analysis of the archaeological context, age, and diets of an array of ancient Cis-Baikal dogs in order to asses how the practice of dog burial relates to the chronology of human mortuary practices, dietary patterns, and subsistence strategies over the course of the last ∼12,000 years. To do this we provide new radiocarbon dates, stable isotope values, and ancient mitochondrial DNA (mtDNA) data for the canid specimens, and integrate previously published data on other regional canids. The contextual information and new technical data point to a series of previously unrecognized geographical and temporal patterns in dog burials in this region, but also a first glimpse at the DNA of early dogs in Northeast Asia. To best understand these datasets, the general culture history of the region is first discussed, including brief notes on the previous identification of dogs from each period, and trends in human dietary and subsistence practices are outlined.

### Background

The Early Holocene in Cis-Baikal, referred to as the Mesolithic, is characterized by the use of microblade technologies and the absence of pottery and cemeteries (Table 1). One wolf burial and one unburied dog have been reported from this period [4]. Forager cemeteries first appear here ∼8000 B.P. at the start of the Early Neolithic period, which also is said to be marked by the use of pottery and ground stone technologies (but not agriculture; [3], [5]). Several dogs have been identified from the Early Neolithic, including burials in cemeteries [2]. Around 7000 to 6800 B.P. the Early Neolithic mortuary traditions cease–nearly no humans are interred here for ∼1000 years. At 6000 to 5800 B.P., with the advent of the Late Neolithic period, human burials again become common, but are of different mortuary traditions. Dog remains have not been reported for the Late Neolithic.

**Table 1:** Simplified Holocene culture history model for Cis-Baikal.

Culture History Period	Approximate Age Range cal. BP	Human Burials	Dog Burials Angara/South Baikal	Dog Burials Priol’khon’e
Mesolithic	12000 to 8000	Rare or absent	Absent	Absent
Early Neolithic	8000 to 7000	Present	Present	Present
Middle Neolithic	7000 to 6000	Rare or absent	Absent	Absent
Late Neolithic	6000 to 5000	Present	Rare or absent	Absent
Early Bronze Age	5000 to 3400	Present	Present	Present
Late Bronze Age	3400 to 2250	Present	Absent	Absent
Early Iron Age	2250 to 1350	Present	Absent	Absent
Late Iron Age	1350 to 850	Present	Absent	Sacrifices only
Early Mongolian	850 to 550	Present	Absent	Absent
Ethnohistoric	550 to present	Present	Absent	Absent

Dog burial trends by culture history period are indicated in the right two columns. Periods prior to the Late Bronze Age are thought to include only foraging groups, with pastoralist arriving around 3400 cal. BP.

The time between the Early and Late Neolithic mortuary traditions has been referred to as the ‘hiatus’ or Middle Neolithic, and genetic research has demonstrated that the latter human populations were genetically discontinuous with the Early Neolithic mortuary populations [6], [7]. No dog remains have been identified from the hiatus period. A third major period of forager cemetery use spans from 5200/5000 to 4000 B.P., or the Early Bronze age, and these groups may be culturally and genetically derived from local Late Neolithic populations. Dog remains previously assigned to this period include burials and isolated elements [2]. After ∼3400 B.P., pastoralists primarily herding sheep, goats, cattle, and horses arrive and establish a suite of new burial traditions [8], [9]. The chronology of Cis-Baikal’s Late Holocene culture history is subdivided here into the Late Bronze Age (∼3400 to 2250 B.P), Early Iron Age (∼2250 to 1350 B.P.), Late Iron Age (1350 to 850 B.P.), and Early Mongolian (850 to 550 B.P.) periods. No dog remains have been described from any of these periods.

Human dietary behaviors during the Holocene in Cis-Baikal have been assessed using stable carbon and nitrogen isotope analyses of human skeletal remains, and through the study of faunal remains from habitation sites. For the Mesolithic, fauna at habitation sites suggest a focus on hunting large ungulates, including deer [10]. Faunal remains and stable carbon and nitrogen isotope values for the region’s Middle Holocene human foragers indicate that these groups relied primarily on terrestrial game such as red deer (*Cervus elaphus*) and roe deer (*Capreolus pygargus*) and increased but variable amounts of the region’s freshwater fauna, including riverine and lake fish and Lake Baikal seal (*Phoca sibirica*) [11]–[16]. Overall, protein diets at this time were least based on aquatic foods among foragers living on the Upper Lena River, and more aquatically focused among those living on the shores of Lake Baikal and on the Angara River. No stable isotope data is available for Cis-Baikal’s Late Holocene human remains, but zooarchaeological data indicates subsistence economies based on herding, hunting (including for deer), sealing, and some fishing, at least along the lake shore [9].

Importantly, the Holocene culture history chronology for Cis-Baikal mostly has been built through radiocarbon dating human skeletal remains, and it is clear that its precision needs to be reevaluated because of the recent documentation of an old carbon offset in Lake Baikal’s aquatic fauna [17]. Humans and dogs that consumed aquatic foods from the lake and its outlet, the Angara River, also likely carry this offset to some extent, and potentially have produced radiocarbon age assessments centuries older than their true ages. While this project was not designed to provide a model for correcting radiocarbon dates on Cis-Baikal dog or human remains, understanding dogs’ dependence on aquatic foods relative to local humans should help to assess the relative extent of bias in the dates made on dog remains, allowing us to more firmly assign them to culture history period.

The canid specimens analyzed here come from two subregions of Cis-Baikal, the Angara River/South Baikal region, and Priol’khon’e (Figure 1). The third Cis-Baikal subregion where extensive archaeological research has occurred, the Upper Lena, has yet to produce clear evidence of dog remains from any period; only a single unidentified and undated canid has been reported [18], but was unavailable to us for study. The Angara River/South Baikal region and Priol’khon’e have quite different ecologies, and these differences in ecology likely shaped local subsistence patterns and stable isotope ecologies. The Angara River is Lake Baikal’s only outlet and is ecologically influenced by the lake, especially in terms of the fishery in its upper sections. Black grayling (*Thymallus arcticus baicalensis*) and lenok (*Brachymystax lenok*), for example, move from the lake into the river as far as Irkutsk for spawning [19]. As the Angara progresses further downstream to the northwest, its fish species take on a more typical Siberia boreal river composition. The surrounding landscape is characterized by rolling hills, a mix of forest types, and in some areas, patches of forest-steppe. The adjacent South Baikal region includes the lake’s south shoreline and the Irkut River valley, which has tributaries that drain several high-altitude mountain ranges. It generally is somewhat wetter than the Angara region, and some areas are densely forested. The second study subregion, Priol’khon’e, which is the area on the west coast of Lake Baikal around the Little Sea, is markedly more arid than first subregion, with steppe and forest-steppe vegetation being dominant. The southern end of the Little Sea, the body of water between Ol’khon Island and the west coast, is one of the lake’s larger stretches of relatively shallow (<5 m) water, and is a rich littoral fishery [20]. Hills, mountains, and cliffs characterize the coastline on Ol’khon’s east coast and that immediately north and south of the Little Sea. These shoreline regions are adjacent to some of the lake’s deepest sections and water temperatures typically are cold, even in summer. Here the density of fishes in the upper portion of the water column generally is low; Baikal seals congregate in these areas of the lake, particularly when it is ice-covered.

## Materials and Methods

### Archaeological and Reference Material

The canid remains analyzed in this study derive from excavations carried out as early as the 1950s. For some individuals, only small element fragments or crania now survive, limiting the extent of analyses possible. Because all specimens analyzed in this study were excavated during other projects, no permits were needed to conduct the analyses reported here. Contextual information for the canid remains was derived from original site reports, publications, and photographs, and is summarized in Tables 2 and 3.

**Table 2:** Context information and radiocarbon dating data for Angara/South Baikal canid remains.

Site/Specimen	Sample #	Context	14C Lab #	Dated material	14C age	+/−	Calibrated age BP(1 sigma)	% collagen yield	Culture history period	Citationfor context
Shamanka II	E2008.175	Primary burial; whole skeleton, within a human grave	Ox20561	Vertebra	6430	35	7420 to 7325	8.3	Early Neolithic	[2]
Lokomotiv wolf sample 1	H2003.704	Primary burial; whole skeleton,interred with disarticulatedhuman remains; human headwith articulated atlas, axis,and mandible betweenthe wolf’s legs	GIN8841a	Ribs and vertebrae	7230	40	8150 to 7980	*	Late Mesolithic/Early Neolithic	[2], [66]
Lokomotiv wolf sample 2	H2003.704		TO11558	Rib	7320	70	8185 to 8030	2.7		
Ust'-Belaia dog 1	2010-004	Primary burial; whole skeleton,articulated; wearing a necklaceand buried with other faunalremains in pit 5, trench 2	Ox23874	Palatine	5981	34	6880 to 6755	11.3	Early/Middle Neolithic	[67], [68]
Ust'-Belaia dog 2	2010-018	Non-burial (?); whole skeleton, partially disarticulated, foundwithin a pit, square 49,trench 2, 140 cm	Ox23875	Rib	6213	33	7175 to 7020	10.6	Early Neolithic	[67], [68]
Ust'-Belaia dog 3	2010-022	Non-burial (?); whole skeleton, partially disarticulated; trench 1,sq. 56, 75 cm; under hearth 2	Ox23877	Rib	5596	34	6410 to 6320	4.4	Middle Neolithic	[67], [68]
Ust'-Belaia dog 4	2010-020	Unknown provenience within site	Ox23876	Rib	5946	32	6845 to 6730	16.9	Early/Middle Neolithic	
Ust'-Khaita	2010-002	Non-burial; disarticulated partial skeleton in layer 9	Ox23873	Nasal	10375	45	12380 to 12135	5.7	Upper Paleolithic	[4]
Pad' Kalashnikova pit 1 dog	2010-024	Primary burial with multiple artifacts; whole skeleton in pit 1	Ox23910	Rib	6122	31	7150 to 6945	16.7	Early Neolithic	[69]
Pad' Kalashnikova pit 2 dog	2010-026	Primary burial; whole skeletonin pit 2	Ox23911	Rib	6075	32	6980 to 6890	10.4	Early Neolithic	[69]

* data not available.

**Table 3:** Context information and radiocarbon dating data for Priol’khon’e canid remains.

Site/Specimen	Sample #	Context	14C Lab #	Dated material	14C age	+/−	Calibrated age BP(1 sigma)	% collagen yield	Culture history period	Citation for context
Bugul'deika II	2010-006	Non-burial; isolated mandible,layer II-2	Ox23878	Mandible	3002	29	3260 to 3085	3.9	Late Bronze Age	This study
Khotoruk	1997.282-2	Burial (?); scattered remains,likely of a wolf, in mid to upperportion of undatedhuman grave 4	Ox23872	Metacarpal 3	1493	24	1395 to 1350	15.1	Early Iron Age	[44]
Uliarba II sample 1	2010-012	Secondary burial; partialskeleton, disarticulated, upperlevel of human grave 35	Ox23879	Mandible	3858	29	4405 to 4185	8.1	Early Bronze Age	[70]
Uliarba II sample 2	2010-012		Ox23880	Mandible	3833	28	4290 to 4155	7.5		
Ulan-Khada	2010-015	Non-burial; disarticulated partialskeleton, upper layer 1	Ox23881	Axis	3995	29	4515 to 4425	5.0	Early Bronze Age	[71]
Shamanskii Mys	1997.281-2	Primary burial; whole skeleton,interred within a pit and withmultiple artifacts, near EarlyNeolithic grave 1, 1973	Ox23870	Ulna	6481	34	7430 to 7330	7.3	Early Neolithic	[44]
Shamanskii Mys	1997.278-1	Primary burial; whole skeletonburied to the right of humanin grave 3, 1972	Ox23871	Mandible	6669	34	7580 to 7510	2.7	Early Neolithic	[44]
Todakta I	2010-008	Sacrifice interment; articulatedwhole skeleton in a pit witha calf skull and feet, possiblyrepresenting a sacrifice	Ox23912	Rib	1062	23	980 to 935	8.0	Late Iron Age	[72]

All newly reported radiocarbon dates are AMS dates obtained through the Oxford Radiocarbon Accelerator Unit [21], [22]. Radiocarbon results are summarized in Tables 2 and 3, and all were calibrated using Oxcal (v4.1) and the INTCAL09 dataset [23]. Site locations are shown on Figure 1.

Where possible, the prehistoric canids from Cis-Baikal were compared with reference sets of recent and prehistoric dogs and wolves. Standard measurements on the Cis-Baikal canid crania, mandibles, dentition, and long bones are presented in Table 4.

**Table 4:** Metric data for Cis-Baikal canids. VDD measurements follow [73], MY measurements are from [74]. Long bone measurements are greatest length, following [73].

Dimension	Shamanka II	Lokomotiv wolf	Ust’-Khaita	Ust’-Belaia dog 1	Ust’-Belaia dog 2	Ust’-Belaia dog 3	Ust’-Belaia dog 4	Pad’ Kalashnikovapit 1 dog	Pad’ Kalashnikovapit 2 dog	Uliarba II	Ulan -Khada	Todakta I
VDD-1	215.9	266.0	193.1	213.0	190.5		165.8	169.0	182.7			
VDD-2	199.5	251.1	181.3	199.1	177.8		157.7	160.6	180.2			
VDD-3	189.2	235.0	171.0	188.0								
VDD-7	104.1	123.9	84.9	106.2	88		76.7	77.2	85.0	92.0		68.8
VDD-8	109.7	139.3	95.8	101.6	93.3		81.3	85.2	95.3			
VDD-9	123.7	159.4	116.4	116.9	112		96.5	100.4	109.0			
VDD-12	94.0	123.7	90.6	71.9								
VDD-13a	105.0	133.6	99.6	104.7	95.3		85.9	86.0	98.0			
VDD-15	71.6	92.0	70.4	71.0	67	65	60.0	63.7	67.5		69.5	57.3
VDD-16	19.9	24.8	21.2	19.4	18.7	17.0	18.0	18.0	18.2	18.9		
VDD-17	55.9	74.3	53.9	54.9	52.0	51.5	47.0	49.7	51.2		53.8	38.5
VDD-18	18.0	26.9	22.1	21.0	20.3	18.5	20.0	19.5	19.0	19.8	19.3	16.6
VDD-20L	15.0	16.7	14.9	13.8	13.4	12.0	12.6	12.8	13.4	13.1	13.1	11.7
VDD-29	60.0	65.8	57.3	55.6	53		53.5	50.7	57.0	51.2		50.8
VDD-30	120.4		100.5	117.2	94.7							
VDD-34	70.7	91.3	72.4	69.2	62.5			59.1	61.5	62.7		
VDD-35	38.1	49.6	43.0	36.1	34.0	33.3	31.5	34.1				
MY-IM2	103.2	141.2	104.1	103.3	98.7		89.3	89.7	99.0			
MY-OI1	94.7	122.9	88.9	89.5	86		73	76.1	84.5			
MY-P3	12.4	17.6	14.9	13.8	12.3		12.3	11.4	12.4	11.2	11.6	
Humerus	181	237			161	153.5	138	139	161		173	117*
Ulna	218	261										
Radius	181	233			156						173	122*
Femur	203	256			172		144		172			137*
Tibia	199	258					144					141*

* Measurements taken by holding unfused epiphyses in place.

The first comparative reference group we utilize consists of European Paleolithic dogs [24], [25] (Tables 5, 6, 7). The second reference group is made up of Chukotka dogs from the 19^th^ and 20^th^ century, hereafter termed Siberian Huskies. The third reference group consists of recent North American Arctic dogs, all from the 20^th^ century. Reference groups of wolves measured by us include Pleistocene (Weichselian) wolves from Belgium, the Czech Republic, the Ukraine and Russia [24], and recent Siberian and Tibetan wolves. From the literature we added Weichselian wolves from southern France [26], recent North American wolves [27], and European Mesolithic dogs from England [28], Germany [29], Portugal [30], and the Baltic region [31]. Estimates of the canid’s shoulder heights, based on their long bone lengths (following [32]), are given in Table 7, also for comparative purposes.

**Table 5:** Comparison of cranial lengths of Cis-Baikal canids with modern and ancient canids.

Specimen/population (n)	Total skulllength/range (mm)	Mean	St. d.
Shamanka II dog	215.9		
Ust'-Belaia 1	213.0		
Ust'-Belaia 2	190.5		
Ust'-Belaia 4	165.8		
Pad' Kalashnikova 1	169.0		
Pad' Kalashnikova 2	182.7		
Lokomotiv wolf	266.0		
European Palaeolithic dogs^a^ (8)	225.7–256.0	235.1	10.15
Recent Siberian Huskies^b^ (18)	168.9–217.0	196.7	14.05
Recent NA Arctic dogs^c^ (16)	202.0–233.0	214.3	16.95
Recent Siberian wolves^b^ (19)	233.4–273.0	248.5	10.08
Recent *Canis lupus chanco* b (8)	221.6–247.5	234.4	8.33
Pleistocene wolves^a^ (6)	253.7–276.5	263.3	8.34

a [24], [25].

b This study, [24].

c This study.

**Table 6:** Comparison of teeth size of the Ust’-Khaita canid with select canid populations. All measurements in mm.

	P^3^ length				P^4^ length				M^1^ length				M_1_ length			
Specimen/population	N	Range	Mean	St.d.	N	Range	Mean	St.d.	N	Range	Mean	St.d.	N	Range	Mean	St.d.
Ust'-Khaita canid	1	14.9			1	22.1			1	14.9			1	26.53		
Paleolithic dogs^a^	5	14.60–17.50	16.28	1.24	8	23.86–27.30	25.23	1.15	6	14.60–19.00	16.69	1.73	2	25.50–29.00	27.25	
Pleistocene wolves^a^	5	16.50–17.93	17.33	0.57	6	25.10–28.60	26.38	1.32	5	15.90–18.57	17.49	1.13	3	30.52–31.70	30.98	0.63
Prehistoric dogs^b^	3	12.80–14.00	13.15	0.6	3	17.20–19.50	18.67	1.27					2	21.6–26.9	24.25	
Baltic Sea Mesolithic dogs^d^					18	16.1–21.1	18.5	1.53					29	19.4–24.8	22.2	1.31
Cis-Baikal Middle Holocene dogs^c^	8	11.2–13.8	12.18	0.82	9	18.0–21.0	19.5	0.92	9	12.0–15.0	13.24	0.84	5	20.63–24.30	21.74	1.47
Recent Siberian wolvesa,c					25	22.82–28.00	24.77	1.47	11	13.97–18.01	15.78	1.17	19	26.70–32.90	29.25	1.77
Recent Canis lupus chancoa,c					8	23.60–26.90	25.01	1.11					8	26.40–31.00	28.71	1.75
Recent Siberian Huskiesa,c					15	17.09–21.50	19	1.05	4	11.11–12.50	11.66	0.59	15	20.60–24.50	22.53	1.19
Recent N.A. Arctic dogs^c^	38	10.90–15.21	13.83	0.93	36	18.18–24.09	21.07	1.19	38	11.72–15.02	13.6	0.76	35	21.30–26.05	24.08	1.33

a This study, [24].

b [28], [29], [30].

c This study.

d [31].

**Table 7:** Comparison of Cis-Baikal canid long bone lengths with those of modern and ancient wolves, and large northern dogs. Shoulder heights are estimates based on regression equations on long bone lengths [32].

Specimen/population (n)	Humerus length/range (mm)	Humerus mean length (mm)	St. d.	Shoulder height (cm)
Shamanka II dog	181.0			60
Ust'-Belaia 2	161.0			53
Ust'-Belaia 3	154.0			50
Ust'-Belaia 4	138.0			45
Pad' Kalashnikova 1	139.0			45
Pad' Kalashnikova 2	161.0			53
Ulan-Khada	173.0			57
Lokomotiv wolf	237.0			79
Recent NA Arctic dogs^a^ (9)	141.2–202.5	182.64	23.01	46–67
Recent NA wolves^b^ (40)		215.66	26.10	
Weichselian wolves^c^ (5)	190.5–222.4	209.40	12.96	63–74
	**Radius length/range (mm)**	**Radius mean length (mm)**	**St. d.**	**Shoulder height (cm)**
Shamanka II dog	181			60
Ust'-Belaia 2	156			52
Ulan-Khada	173			57
Lokomotiv wolf	233			76
Recent NA Arctic dogs^a^ (10)	137.1–196.0	170.76	19.75	46–64
Recent NA wolves^b^ (40)		210.53	17.83	
Weichselian wolves^c^ (7)	206.1–222.6	214.76	5.42	68–73
	**Femur length/range (mm)**	**Femur mean length (mm)**	**St. d.**	**Shoulder height (cm)**
Shamanka II dog	203			62
Ust'-Belaia 2	172			52
Ust'-Belaia 4	144			44
Pad' Kalashnikova 2	172			53
Lokomotiv wolf	256			79
Recent NA Arctic dogs^a^ (12)	150.5–217.5	197.54	20.88	46–67
Recent NA wolves^b^ (40)		229.78	22.40	
Weichselian wolves^c^ (4)	219.7–242.9	225.5	10.06	68–75
	**Tibia length/range (mm)**	**Tibia mean length (mm)**	**St. d.**	**Shoulder height (cm)**
Shamanka II dog	199			59
Ust'-Belaia 4	144			43
Lokomotiv wolf	258			76
Recent NA Arctic dogs^a^ (12)	151.6–212.5	191.50	19.48	45–63
Recent NA wolves^b^ (40)		234.31	22.38	
Weichselian wolves^c^ (9)	215.1–245.3	225.33	12.26	64–73

a This study.

b [27].

c [26].

### Genetic Analyses

Bone preparation, DNA isolation and polymerase chain reaction (PCR) set-up were all performed in a dedicated, spatially isolated ancient DNA laboratory using all standard ancient DNA precautions. Negative controls were included with each extraction and PCR. DNA extraction was either with phenol-chloroform as described in [2] or with the silica column based DNeasy kit (Qiagen). Fragments of the mitochondrial DNA were amplified in 25 µl reactions including 3 µl of extract. Reactions contained 1× buffer, 2.5 mM MgCl2, 0.2 mM dNTPs, 1 µM of each primer and 2.5 U AmpliTaq Gold (Applied Biosystems). Primers which target the ca. 425 base pairs at the 5′ end of the mitochondrial control region in three overlapping fragments were used from [33] (size uncertainty due to presence of indels). The PCR conditions were an initial 5 min denaturation step at 95°C followed by 20 cycles of 95°C for 30 s, touchdown 0.5°C/step from 60 to 50 for 1 min, and extension at 72°C for 1 min followed by 40 cycles of 95°C for 30s, 48°C for 1 min and 72°C for 1 min with a final elongation step of 7 min at 72°C, following [2]. Amplification was attempted from each extract at least twice for each fragment. All PCRs included negative PCR controls and the extraction negative controls. All reactions were checked on agarose gels stained with CyberSafe (Invitrogen), and all bands were directly (Sanger) sequenced in both directions with the same primers as were used in the PCR.

A BLAST search (http://www.ncbi.nlm.nih.gov/genbank) was made with each clean sequence to determine taxa of origin, to ensure it was canid and not a common reagent contaminant such as human or cow [34]. Sequences of canid origin were checked against replicates of the same fragment and overlapping fragments, and concatenated in Geneious [35]. Degredation via deamination of the DNA in ancient material can yield apparent mutations. These should occur randomly, so they can be identified and resolved through replication. In the two cases where a mismatch was identified between replicates, the base pair was resolved through generation of sequences from at least one additional independent amplification. Sequences were aligned with a representative subset of previously published dog and wolf sequences [2], [33], [36], [37], [38] in MUSCLE [39] and a phylogenetic analysis was performed using a maximum likelihood algorithm and an HKY85 model of sequence evolution (as in [2]), four rate categories, and an estimated gamma parameter and transition/transversion ratio in the program PHYML [40]. Coyote (*Canis latrans*) haplotypes were used to root the phylogeny [37]. The minimum spanning network of the dog clade I haplotypes, as identified in the phylogeny, was constructed using TCS v. 1.21 [41].

### Stable Isotope Analyses

The stable carbon (δ^13^C) and nitrogen (δ^15^N) isotope ecology of Cis-Baikal was previously established through the analyses of 251 modern and archaeological faunal specimens [11]–[15]. These analyses were conducted at the University of Calgary, using the methods outlined in [14]. Briefly, samples were cleaned ultrasonically, demineralized in 1% HCl, and soaked in 0.125 NaOH before being freeze-dried, ground and analyzed. The new canid stable isotope values presented below were obtained through the Oxford Radiocarbon Accelerator Unit using the methods outlined in [21]. These were the same samples utilized for radiocarbon dating, and the C/N ratios reported in Tables 2 and 3 were used to help evaluate sample preservation for both the radiocarbon dates and the stable isotope results. For analyses, these samples were ground, demineralized in 0.5 M HCl and treated with 0.1 M NaOH before being gelatinized and ultrafiltered to collect the >30-kD gelatin fraction, which was freeze-dried and analyzed. To test for inter-comparability of human and canid stable isotope results, three canid samples were analyzed by both laboratories, with the average difference in δ^13^C being 0.4‰, and δ^15^N 0.13‰.

## Results

### Osteometric Identification

Seven complete adult canid skulls are available from Holocene sites in Cis-Baikal. The Shamanka II dog skull and the Lokomotiv wolf skull were studied previously [2]. The five remaining complete skulls, three from Ust’-Belaia and two from Pad’ Kalashnikova, have lengths ranging from 165.8–213 mm (Tables 4, 5). They are all shorter than the skulls of the recent and Pleistocene wolves in our reference groups. The Cis-Baikal skulls fit in the size range of the recent Siberian husky skulls, except for the Ust’-Belaia 4 skull, which is shorter than these modern specimens (Table 5). Except for the Ust’-Belaia 1 skull, all are shorter than those of the recent North American Arctic dogs, and all five are shorter than those of the European Palaeolithic dogs. The upper carnassials of the five Cis-Baikal skulls (Table 4) are comparable in length to the carnassials of the recent dogs in our data set and are clearly smaller than the carnassials of the fossil and recent wolves (Table 6). In other words, all this size data points to the specimens being dogs, which is unsurprising, given that all date to Middle Holocene, long after the initial domestication of dogs had began.

**Figure 2:**
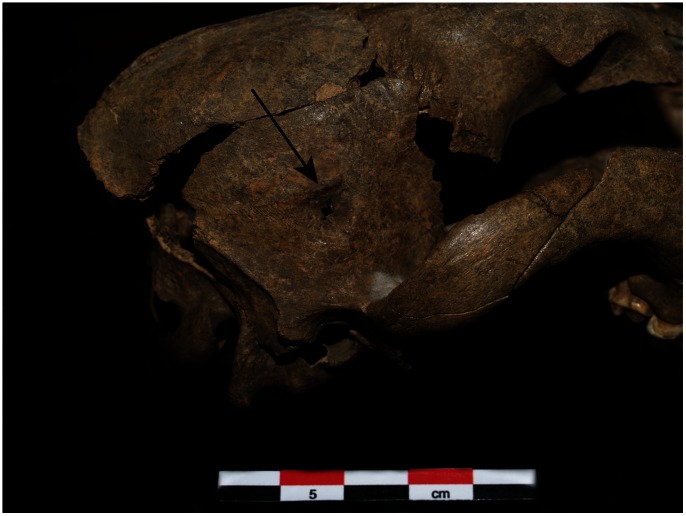
The cranium of the Ust’-Khaita canid with the location of the partially healed puncture to the cranium indicated.

While the cranium of the Ust’-Khaita canid was mostly complete, it was a juvenile at the time of death, likely 5–8 months of age, making it difficult to compare the shape and size of its cranium to adult specimens of known taxonomic status. All its permanent teeth are fully erupted, and we thus focus on the sizes of its teeth in our attempts to identify this specimen (Table 6). The length of the P^3^ falls inside the ranges of the P^3^ lengths of Palaeolithic dogs and recent North American Arctic dogs. This premolar is slightly longer than the P^3^ from the Cis-Baikal Middle Holocene dogs and European prehistoric dogs, but it is clearly smaller than those of the Pleistocene wolves. The length of the upper carnassial of the Ust’-Khaita canid falls in the range of the tooth lengths of the recent North American Arctic dogs, outside the range of the other dogs, but is smaller than the upper carnassials of all wolves, fossil and recent, in our data set. The upper first molar length falls inside the range of the lengths of this tooth in the European Palaeolithic dogs, the Cis-Baikal Middle Holocene dogs and the recent North American Arctic dogs; it is somewhat longer than this molar of recent Siberian Huskies. It falls just inside the range of recent Siberian wolves, but is smaller than this tooth in the Pleistocene wolves. Finally, the length of the lower carnassial of the Ust’-Khaita canid is similar to that of Palaeolithic dogs and European prehistoric dogs, but is longer than that of the other prehistoric and recent dogs. It falls at the lower limit of the range of the Tibetan wolves but is clearly smaller than this tooth of Pleistocene wolves and recent Siberian wolves. Its post-canine teeth also exhibit crowding, a trait seen elsewhere in early dogs that is relatively rare in wolves, but this could be due to its cranium and mandible not being fully developed [42], [43]. Perhaps also relevant to its identification is the fact that its cranium was punctured near the suture between the right parietal and temporal bones (Figure 2); this partially healed prior to death, perhaps indicating it had received care from humans. Also, the blade of the right ilium was fractured and it too had partially healed. It is possible both injuries occurred in the same event. Given this life history and size data, we only identify this canid as a *probable dog*. Note that the original analysis of this specimen also identified it as a dog [4]. Clearly, however, additional analyses will be needed in the future to more specifically identify this specimen.

Four fragmented canid crania are available from Cis-Baikal, including specimens from Ust’-Belaia, Uliarba II, Ulan-Khada and Todakta I. However, the lengths of their carnassials are clearly shorter than those of all the wolves from our reference groups, and are similar to the lengths of the dog carnassials (Tables 4, 6). Furthermore, the size of the long bones of several of these canids fall in the range of North American Arctic dogs and are clearly smaller than the same elements in wolves (Table 7). Based on the size of their carnassials and long bones, these canid remains also are identified as dogs.

The Bugul’deika canid is represented by only a single fragmented mandible, but its carnassial is shorter than that of all of the other Cis-Baikal canids, except that of the Late Iron Age Todakta I dog, and is clearly far shorter than any wolf carnassials in our dataset (Tables 4, 6). We thus identify this specimen as a dog. Small postcranial fragments are all that remain from the Shamanskii Mys canid skeletons, but the original site reports identify all of these specimens as dogs that were similar in size and shape to modern Siberian Huskies [44]; identification methods are not provided. For this paper we also consider these specimens as dogs. Finally, the Khotoruk canid was represented only by fragments of postcranial elements and was not previously analyzed; it could not be more specifically identified through osteometric analyses.

### Genetic Analyses

**Figure 3:**
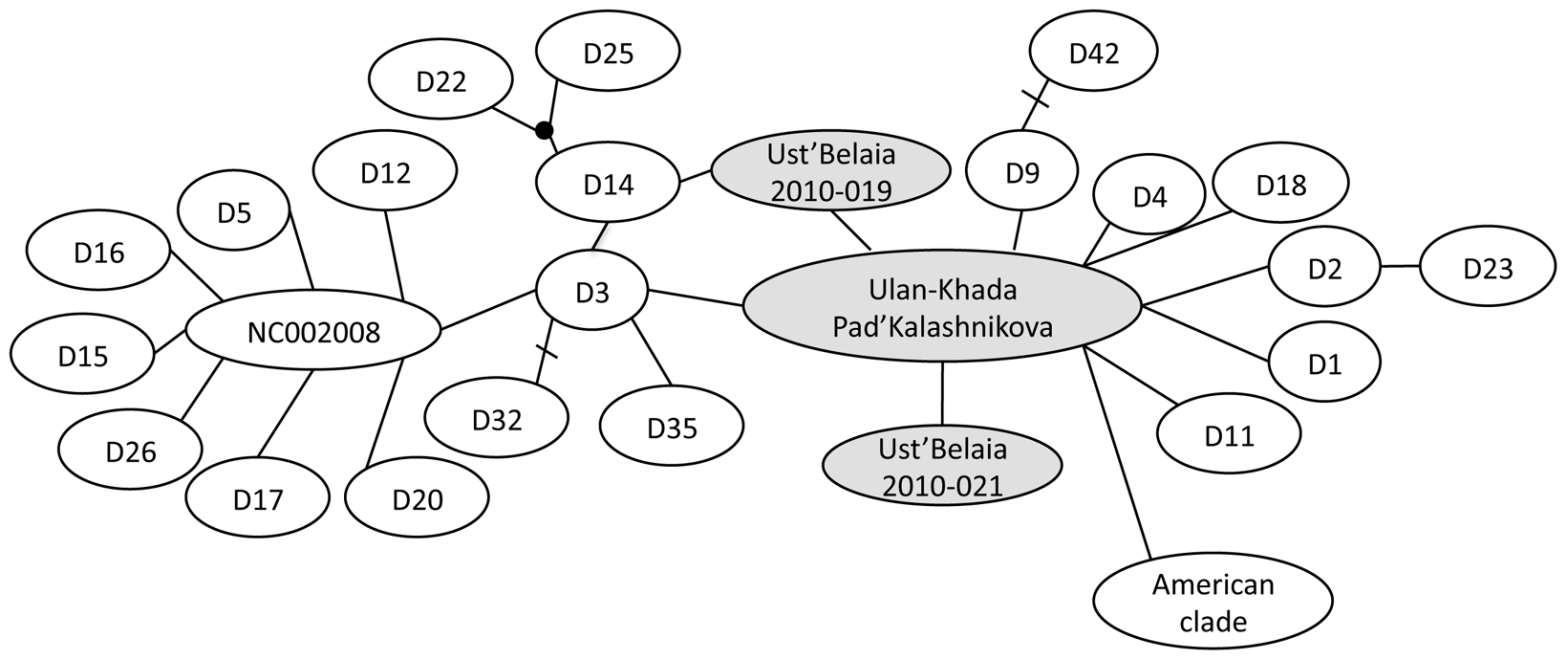
Minimum spanning network of haplotypes in dog clade I. Previously published sequences are in white and the ancient Baikal dogs presented here are in the shaded ovals. The American clade has been collapsed and is represented by the oval labeled “American clade”. Each link represents a single mutation, and bars across them additional mutations. The black circle represents a hypothetical haplotype. Not all alternative links are shown.

**Figure 4:**
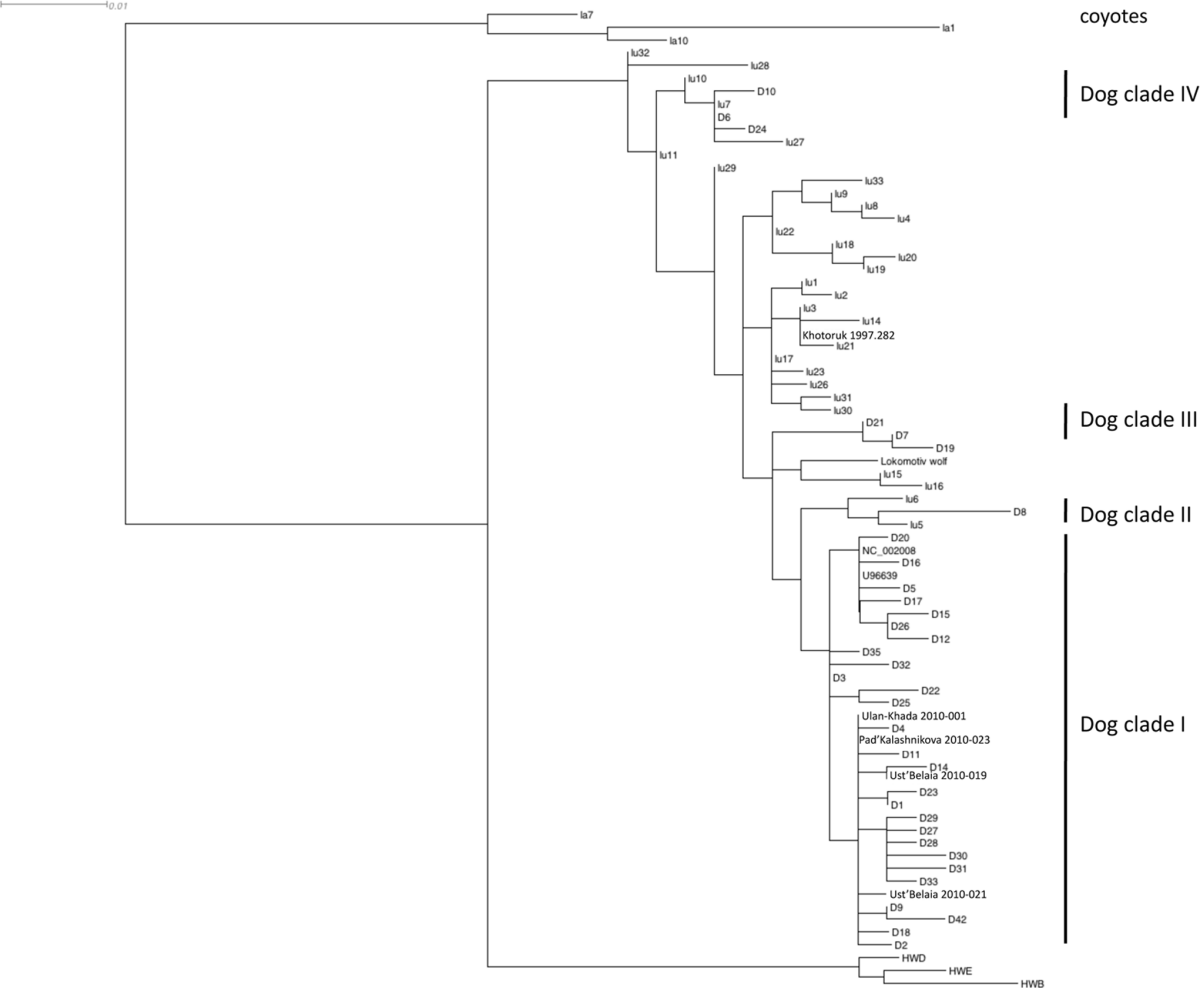
Maximum likelihood phylogeny of recent and ancient dogs and wolves (prefix lu), rooted with coyotes (prefix la). The dogs form the previously identified four clades (labeled dog clades I–IV) within the diversity of wolves. Specimen 2010-19 is Ust’-Belaia dog 2 in the text, 2010-021 is Ust’-Belaia dog 3, 2010-023 Pad’ Kalashnikova dog 1, 1997.282 Khotoruk, and 2010-001 Ulan-Khada.

Of the 14 canid remains analyzed, complete sequences were obtained from five individuals: two from Ust’-Belaia (2010–021 and 2010-019), and one each from Ulan-Khada (2010-001), Pad’ Kalashnikova (2010-023) and Khotoruk (1997.282), and those sequences have been submitted to GenBank (KC776175-KC776179). The first four of these came from remains that were osteometrically identified as dogs and were either the same as a dog in Genbank, or one base pair different. The animals from Ulan-Khada and Pad’ Kalashnikova had the same haplotype, which is widespread and common in living dogs, and has been documented in America (GenBank accession HQ126706; [45]) and Africa (GQ375187; [46]). The two dogs from Ust’-Belaia had sequences that differed from this common sequence by one mutation, and which were not present in the data base (Figure 3). All three of the dog haplotypes identified here belong to dog clade I, the most common and diverse clade (Figure 4). The last individual, from Khotoruk, was represented by fragments only and could not be osteometically identified. It had a sequence that matched a Russian wolf sequence (GenBank accession GQ376507), and its isotopic signature is consistent with this identification (see below), so we identify it as a wolf.

### Temporal Variability in Dog Burials

Several patterns emerge when examining the radiocarbon dates and contextual information for the Angara River/South Baikal canids (Tables 1, 2, 3). First, the earliest possible dog in this subregion is at Ust’-Khaita, a habitation site, and this animal was not buried upon its death. Three previous radiocarbon dates on unidentified animal bones suggested the layer containing the canid skeleton dated to the Early Holocene [4], [10]. However, a radiocarbon date (Ox23873; 10375±45) on a fragment of the canid cranium indicates it died around 12,380 to 12,135 B.P., at the end of the Pleistocene. Notably, all other faunal remains from the layer, which include specimens of Bovidae, Cervidae, and Equidae, consist of scattered and fragmented skeletal elements. The canid is the only animal in the layer represented by a nearly whole skeleton. If these canid remains truly are from a dog, it would be among the earliest yet documented in East Asia, with specimens identified as dogs and of similar age previously being reported in China and Kamchatka [47]; a far older probable dog recently has been documented in the Altai region west of Cis-Baikal [48], [49].

**Figure 5:**
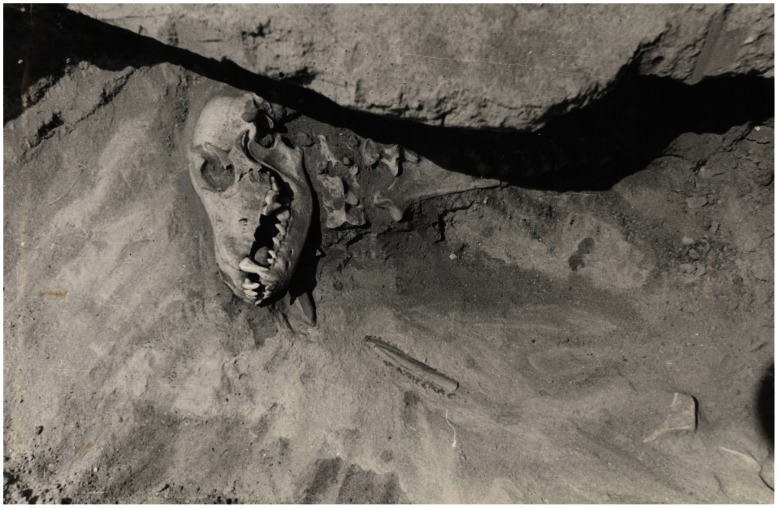
Pad’ Kalashnikova dog from pit #2 under excavation. Stone and bone implements are present near and under the cranium, and a round pebble is visible within the mouth.

**Figure 6:**
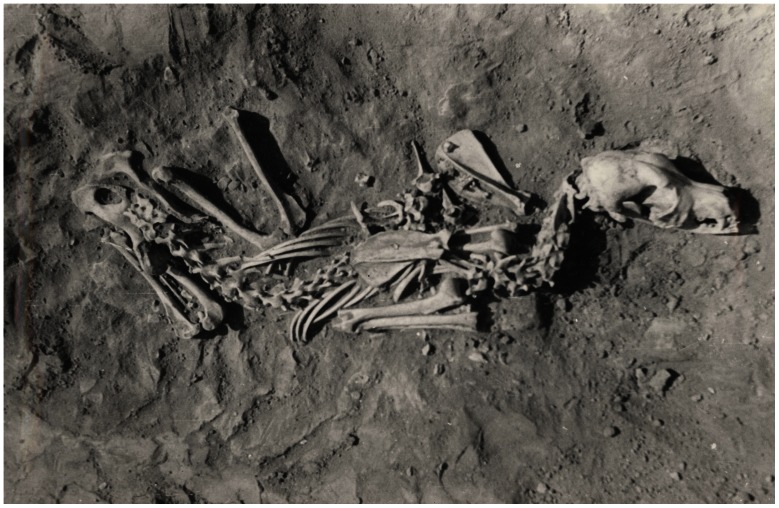
Pad’ Kalasnikova dog from pit #1. This dog was buried in a crouched or sitting position.

**Figure 7:**
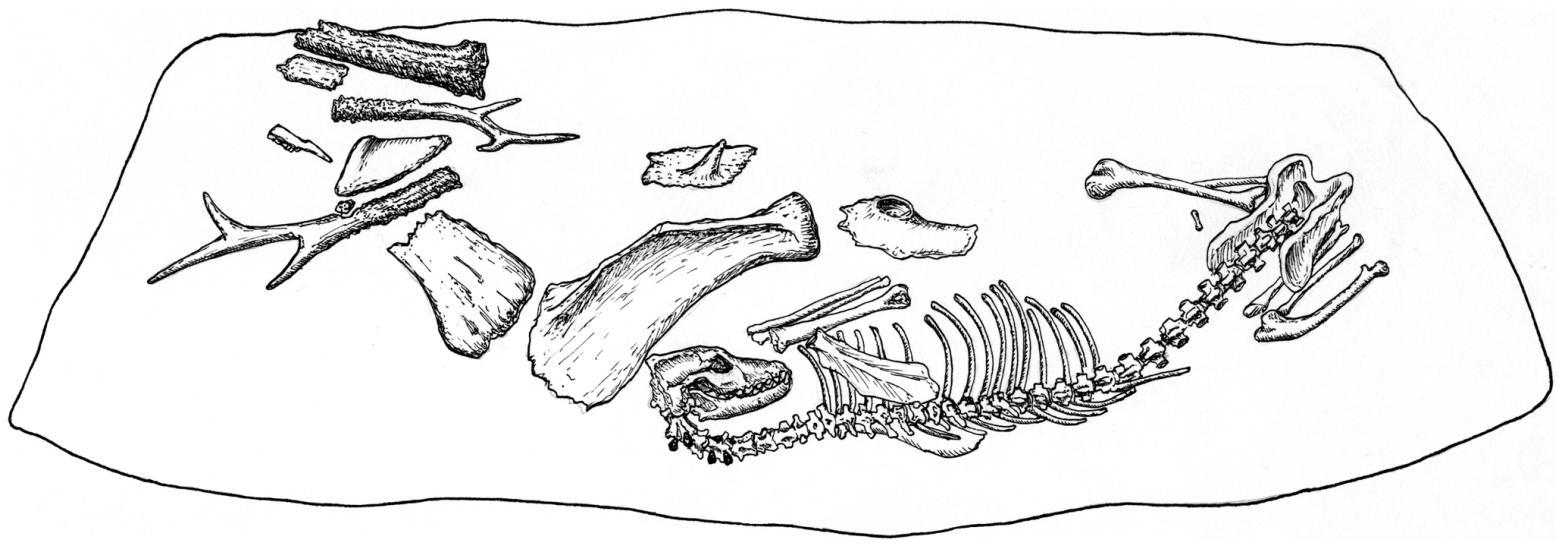
Illustration of dog burial #1 at Ust’-Belaia. The dog was interred wearing a necklace of four red deer canine teeth pendants and placed beside its body were various faunal remains. Redrawn from [67].

Second, canid burials first appear in Angara River/South Baikal subregion near the Mesolithic/Early Neolithic transition, beginning with the wolf interred in the Lokomotiv cemetery (Tables 1, 2). Dog burials from the Early Neolithic are well evidenced and include the previously described whole skeleton in the Shamanka II cemetery [2], and the two primary burials at Pad’ Kalashnikova, a site which contains both habitation site materials and human burials. Multiple artifacts were buried with the body of the pit #2 dog at Pad’ Kalashnikova, and a pebble was placed in its mouth (Figure 5). The second dog burial at this site was interred in the sitting or crouched position but was not associated with artifacts (Figure 6). Perhaps the most interesting dog burial was found in the Ust’-Belaia habitation and cemetery site. This dog (Ust’-Belaia dog 1) was interred wearing a necklace of eight red deer canine tooth pendants and also associated with its skeleton were a Bovidae scapula and horn core, two whole roe deer antlers, and other unidentified bones (Figure 7); this dog appears to have been buried during the Early to Middle Neolithic transition. Ust’-Belaia also contained one dog dating to the Early Neolithic which appears to have been discarded in a trash pit, and one dog of unknown provenience within the site also dating to this period. In addition, one dog dating to the Middle Neolithic was present in Ust’-Belaia, this one also apparently discarded in a pit and found partially disarticulated. Third and finally, our studies provide no evidence for dog burials during the Middle Neolithic, and also absent are dog remains from any contexts dating to the Late Neolithic or later periods in this subregion.

**Figure 8:**
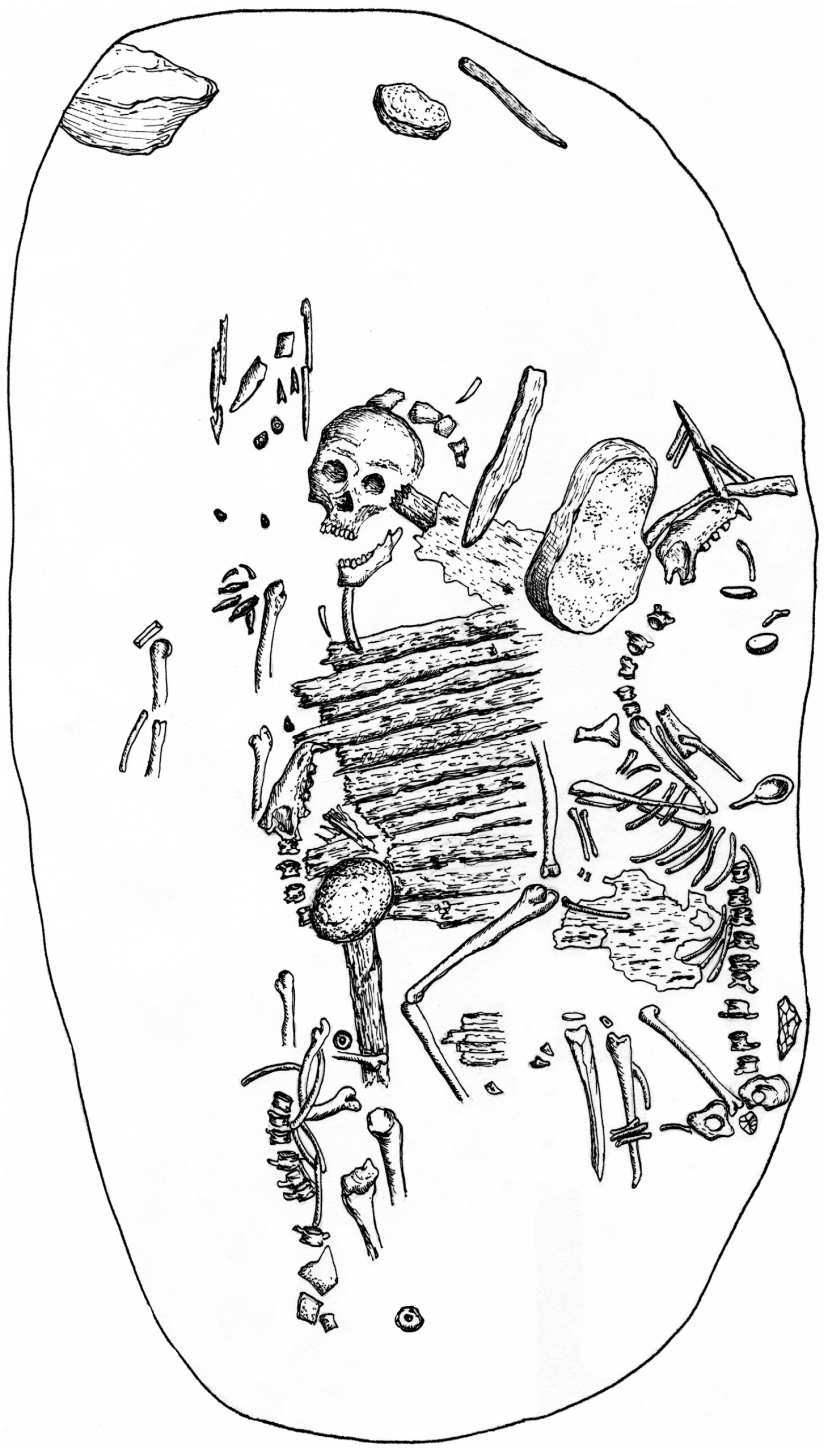
Illustration of grave #3 (1972) at Shamanksii Mys. Figure shows the skeleton of one dog placed above and to the right of the human burial, and a second dog skeleton present to the left of the human body. Note that the sewn birch bark sheet separating the human remains and the dog skeletons is not visible in this illustration, but several of the birch poles that were below this sheet are depicted on the chest of the underlying human burial. Redrawn from [44].

The temporal variability in dog burial practices in Priol’khon’e follows a similar pattern to that seen in the South Baikal/Angara River region, but also provides some indication of dog postmortem treatment during the latter portions of the Holocene (Tables 1, 3). First, the earliest canid burials in Priol’khon’e are found at the Shamanskii Mys habitation and cemetery site, and these date to the Early Neolithic period. These include two primary burials (only one of which is directly dated) of dogs within an elaborate grave containing the remains of a human adult male (which also is directly dated to the Early Neolithic; Figure 8), and a single primary interment of a probable dog in its own pit near another human grave. This latter dog burial also contained multiple artifacts and fragmented faunal remains.

**Figure 9:**
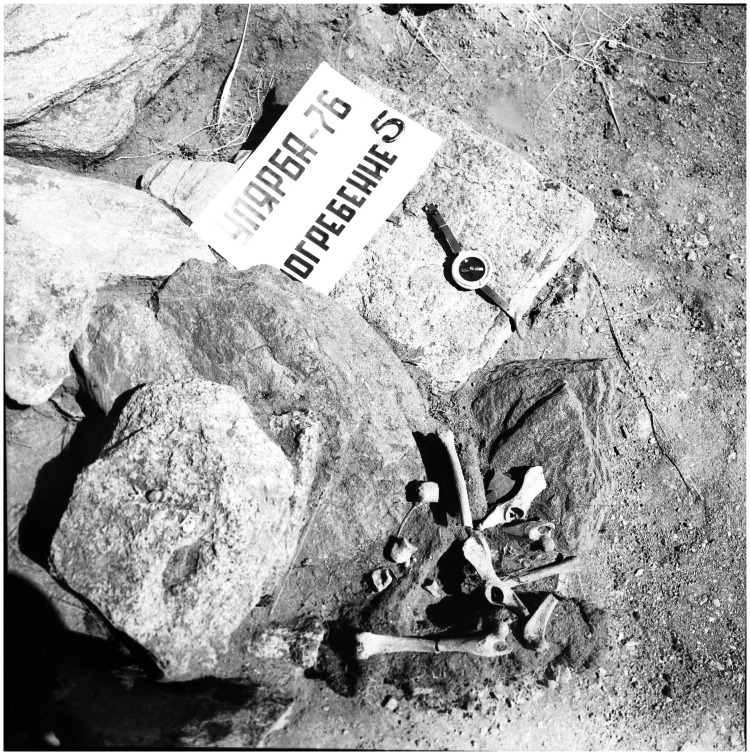
Remains of a secondary dog burial within the upper portion of a human grave at Uliarba II.

**Figure 10:**
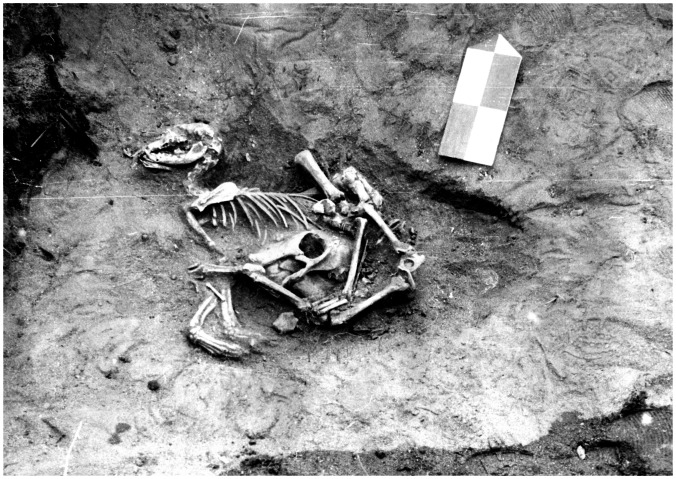
The Todakta I dog interment under excavation [72]. Just below the dog were the cranium, mandible and lower leg bones of a calf. These calf remains may have been left attached to the hide and used to wrap the dog, which appears to have been a sacrificed animal.

Second, dog remains from any context are absent from the Middle and Late Neolithic periods in Priol’khon’e, but are evidenced in later periods. A secondary dog burial was found within the upper portion of a human grave at the Uliarba II cemetery (Figure 9), and like the human remains at this site, dates to the Early Bronze Age. Scattered and unburied remains of another Early Bronze Age dog were found at the Ulan-Khada habitation site, while an isolated dog mandible dating to the Late Bronze age was present at the Bugul’deika II habitation site. The scattered post-cranial remains of the wolf found within the upper portions of an undated grave at the Khotoruk cemetery proved to date to the Early Iron Age, far post-dating the radiocarbon dated human remains at this site, all of which are from the Early Neolithic. Third and finally, the dog from the Todakta I cemetery dates to the Late Iron Age, and this primary burial was found within its own pit near an undated human grave typologically assigned to the Early Mongolian period (Figure 10). Directly under the skeleton of this juvenile dog were the cranium, mandibles, front lower limbs, and caudal vertebrae of a juvenile cow, and smaller fragments of sheep or goat bone. This group of remains may be from a sacrifice, where a young dog was killed and buried with remnants of several eaten animals, including perhaps the hide of the cow with the head and feet still attached.

### Stable Isotope Analyses

**Figure 11:**
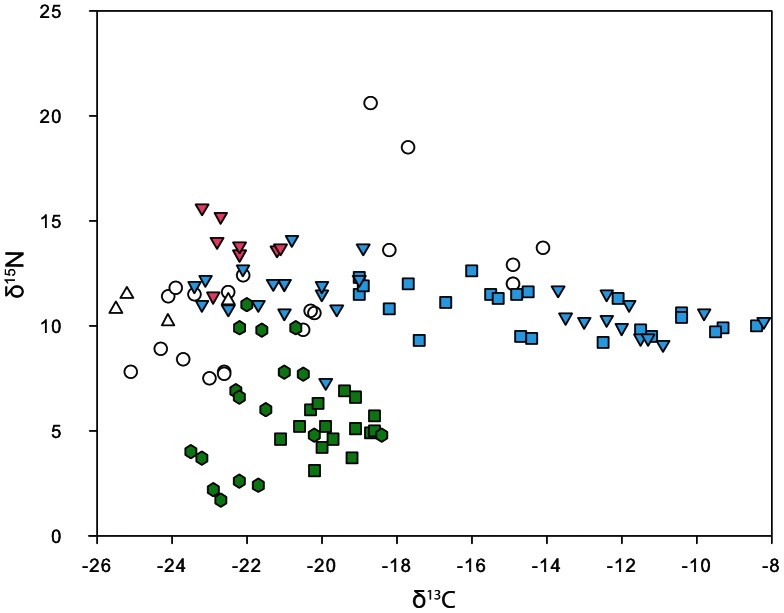
Plot of δ^13^C and δ^15^N values for select Cis-Baikal mammals and fish. Green squares represent archaeological ungulates; green hexagons are modern Eurasian ground squirrel and hare; white circles are modern Angara fish; blue triangles are modern fish from the open Baikal coast; blue squares modern fish from the Little Sea of Lake Baikal; white triangles modern Lena River fish; pink triangles archaeological Baikal seals. Modern samples are adjusted by 1.0‰ on the carbon scale to compensate for the industrial isotope effect.

The stable isotope ecology of Cis-Baikal is well documented but quite complex (Figure 11). In general, fish and Baikal seal here are more enriched in the heavier nitrogen isotope (δ^15^N mean for arch. seal = 13.79±1.19‰; modern fish = 11.08±2.02‰) than terrestrial mammals such as deer (δ^15^N mean for archaeological specimens = 5.14±1.05‰). Variability in the nitrogen isotope values of humans and canids thus indicates the relative contribution of aquatic foods to their diets. Fish in Lake Baikal range widely on the δ^13^C scale (−28.6‰ to −9.6‰), reflecting variability in algae at the base of the aquatic food web. That variability results from different sources of carbon (dissolved organic and inorganic carbon as well as atmospheric CO_2_) and variation in photosynthesis related to water temperature and pH [11], [50]. Fish from shallower waters tend to be enriched in the heavier isotope of carbon relative to fish from open waters. While there is some evidence for a trophic level effect in Baikal of about 1‰ for δ^13^C, the majority of variation in δ^13^C appears to relate to where the fishes were foraging within the lake, not their trophic level. Riverine fish, by contrast, show little variation in δ^13^C since multiple carbon sources are constantly mixed. Human and canid variability on the δ^13^C scale should then relate primarily to the relative reliance upon fish from these varying environments.

**Figure 12:**
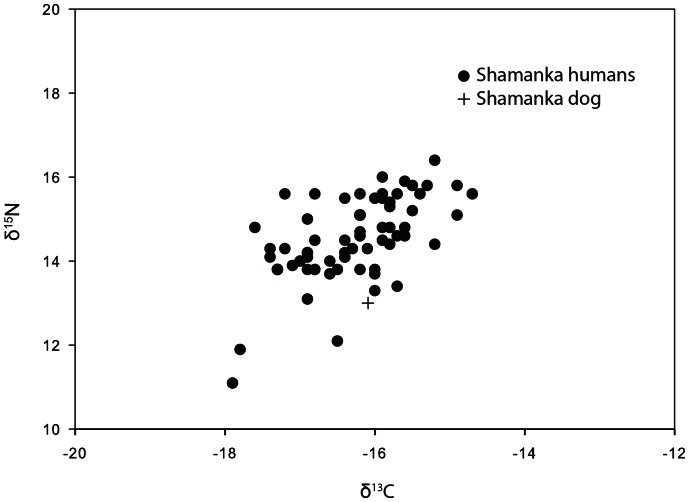
Plot of δ^13^C and δ^15^N values for humans and a dog buried at the Shamanka II cemetery.

**Figure 13:**
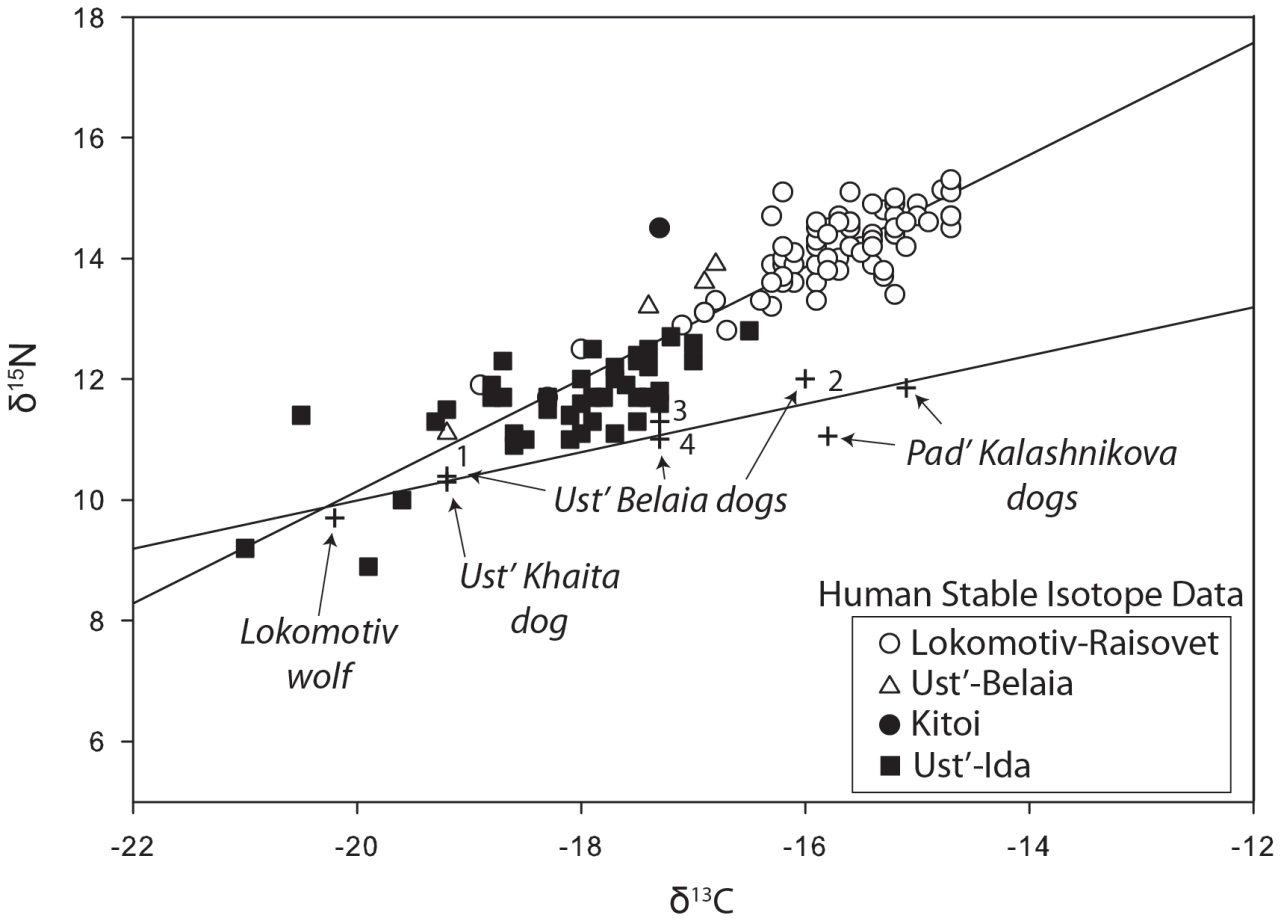
Plot of δ^13^C and δ^15^N values for humans and canids from the Angara River area. Linear regression for human values (y = 0.929x +28.692, R2 = 0.846; N = 119) and canid values (y = 0.399x +17.958, R2 = 0.864; N = 8) indicated.

**Figure 14:**
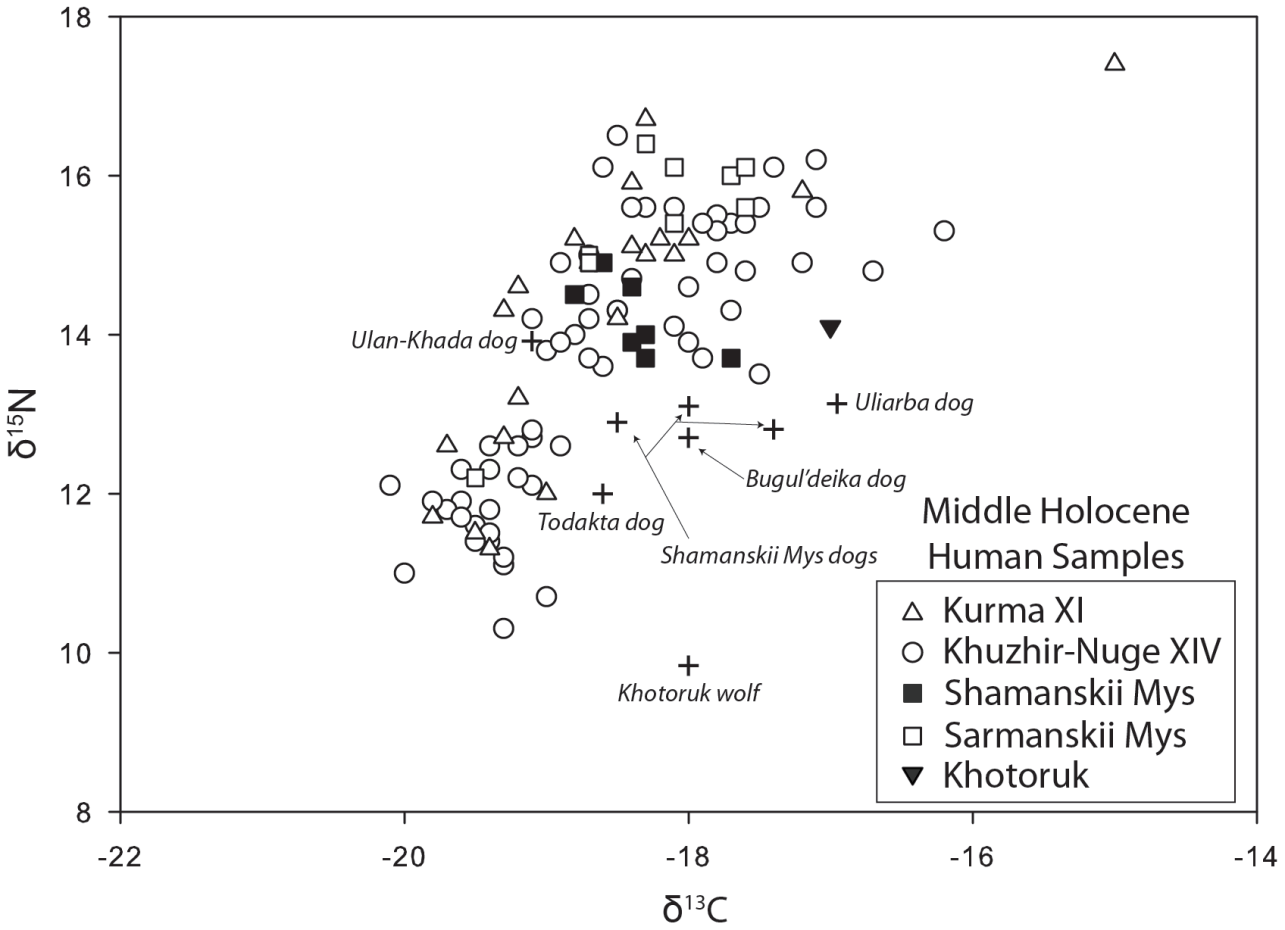
Plot of δ^13^C and δ^15^N values for humans and dogs from the Priol’khon’e area.

Stable isotope values also are available for 282 Middle Holocene human skeletons from South Baikal (n = 62), the Angara River (n = 119) and Priol’khon’e (n = 101; [14], [15]; Figures 12, 13, 14). Previous studies have reported stable isotope values for six Cis-Baikal canids (including two from Shamanskii Mys and one from Khotoruk, also analyzed here), all of which at that time were undated, and three (from Khotoruk, Sagan-Zaba II, and Obkhoi) never identified in the original site reports as specifically dog or wolf [14], [15]. Two canids in these earlier studies (from Sagan-Zaba II and Obkhoi) are excluded here because they remain undated and unidentified. We include the Khotoruk canid isotope values because we were able to genetically identify this specimen as a wolf (see above), and also discussed here are the isotope values from the three Shamanskii Mys canids, all previously identified as dogs in the literature [44]. All canid stable isotope values used here are listed in Tables 8 and 9.

**Table 8:** Stable carbon and nitrogen isotope values for Angara River/South Baikal area canids analyzed in this study.

Site	Sample #	Period	Element	C/N	% collagenyield	δ^13^C	δ^15^N	Citation forisotope values
Shamanka dog	E2008.175	Early Neolithic	Vertebra	3.1	14.2	−16.1	13.0	[1]
Lokomotiv wolf	H2003.704	Late Mesolithic	Rib	3.2	14.3	−20.2	9.7	[1]
Ust’-Belaia dog 1	2010-004	Early/Middle Neolithic	Palatine	3.2	11.3	−19.2	10.4	This study
Ust’-Belaia dog 2	2010-018	Early Neolithic	Rib	3.2	10.6	−16.0	12.0	This study
Ust’-Belaia dog 3	2010-020	Middle Neolithic	Rib	3.2	16.9	−17.3	11.3	This study
Ust’-Belaia dog 4	2010-022	Early/Middle Neolithic	Rib	3.2	6.1	−17.3	11.0	This study
Ust’-Khaita canid	2010-002	Upper Paleolithic	Nasal	3.3	5.7	−19.2	10.3	This study
Pad’ Kalashnikova pit 1 dog	2010-024	Early Neolithic	Rib	3.2	16.7	−15.1	11.9	This study
Pad’ Kalashnikova pit 2 dog	2010-026	Early Neolithic	Rib	3.2	10.4	−15.8	11.1	This study

**Table 9:** Stable carbon and nitrogen isotope values for Priol’kon’e canids analyzed in this study.

Site	Sample #	Period	Element	C/N	% collagenyield	δ13C	δ^15^N	Citation forisotope values
Bugul’deika II dog	2010-006	Late Bronze Age	Mandible	3.3	3.9	−18.0	12.7	This study
Khotoruk wolf	1997-282-2	Early Iron Age	Metacarpal 3	3.2	15.1	−18.0	9.8	This study
Uliarba II dog (2 samples)	2010-012	Early Bronze Age	Mandible	3.3	7.5	−17.0	13.2	This study
Ulan-Khada dog	2010-015	Early Bronze Age	Axis	3.3	5.0	−19.1	13.9	This study
Shamanskii Mys Pit 1 dog burial (1973)	1997.281-2	Early Neolithic	Ulna	3.2	7.3	−17.4	12.8	This study
Shamanskii Mys, Grv. 3 (1972) Right dog	1997.278-1	Early Neolithic	Mandible	3.2	2.7	−18.0	13.1	This study
Shamanskii Mys, Grv. 3 (1972) Left dog	1997.279	Early Neolithic		3.3	20.5	−18.5	12.9	[14], [15]
Todakta I dog	2010-008	Late Iron Age	Rib	3.2	8.0	−18.6	12.0	This study

Moving to results by subregion, the human stable isotope values at the Shamanka II cemetery in South Baikal, all from Early Neolithic individuals, have relatively high δ^13^C and δ^15^N values (Figure 12; δ^13^C mean = −16.27±0.7‰; δ^15^N mean = 14.55±1.0‰), indicating diets with substantial aquatic components from the lake [15]. The Early Neolithic dog buried at this site has a δ^13^C value (−16.1‰) very near the site mean, and a δ^15^N value (13.0‰) well below the site mean (Figure 12). This pattern, where dogs are similarly enriched in δ^13^C and lower in δ^15^N than local humans eating aquatic food-rich diets, generally occurs across all of our study samples.

On the Angara River, human stable isotope values are available for Early and Late Neolithic and Early Bronze Age humans, and these fall into a clear linear pattern when plotted (y = 0.929x +28.692, R^2^ = 0.846; n = 119; Figure 13). The Early Neolithic humans from Lokomotiv (n = 72) are more enriched in δ^13^C and δ^15^N (δ^13^C mean = −15.75±0.8‰; δ^15^N mean = 14.15±0.7‰; Figure 13) while the Late Neolithic and Early Bronze Age individuals from Ust’-Ida (n = 41) about 115 km downstream are less enriched in both isotopes (δ^13^C mean = −18.14±0.9‰; δ^15^N mean = 11.60±0.8‰). Ust’-Belaia is located between these two cemeteries on the Angara River, and the stable isotope values for four humans buried there (three Early Neolithic, one Early Bronze) mostly plot between those of the two larger cemetery samples. Values for one Early Bronze Age Lokomotiv human and one Early Neolithic individual from the Kitoi cemetery also are available. Overall, there is a clear geographical and temporal trend in human diets on the Angara during the Middle Holocene: Early Neolithic individuals’ diets had greater aquatic content than did the diets of Late Neolithic or Early Bronze Age groups, and diets generally were less aquatic as one moved downstream from Lake Baikal.

The canid stable isotope values from the Angara region include those from dogs dating to the Upper Paleolithic/Mesolithic transition, and the Early and Middle Neolithic periods, as well as the Lokomotiv wolf, which dates to the very late Mesolithic (Figure 13). The early Ust’-Khaita probable dog, the Lokomotiv wolf, and the Ust’-Belaia dog buried wearing a necklace all have relatively low isotope values, indicating relatively low aquatic content in their diets. The remaining Angara dogs, including buried and non-buried individuals, have higher δ^13^C and δ^15^N values; the higher δ^15^N values suggest their diets included relatively greater quantities of aquatic foods. The stable isotope values for all the canids fall in a linear pattern when plotted (y = 0.399x+17.958, R^2^ = 0.864; n = 8), and this pattern differs from that seen in the Angara human isotopes values. Specifically, as canid and human isotope values increase in δ^13^C, the canids tend to show lower δ^15^N when compared to humans with the same δ^13^C values.

For Priol’khon’e, stable isotope values are available for four humans dating to the Early Neolithic, with the remainder being Late Neolithic (n = 8) and Early Bronze Age (n = 89; [14], [15]; Figure 14). These data indicate substantial variability in reliance upon aquatic foods from the lake, with δ^15^N values ranging from 10.3‰ to 17.4‰. The δ^13^C values range from −20.1‰ to −16.9‰, with one individual with a δ^13^C value of 15.0‰ identified previously as an outlier [15]. While clearly less linear than the Angara River human dataset, the Priol’khon’e human δ^13^C and δ^15^N values are still positively correlated (with outlier removed, R^2^ = 0.542; y = 1.485*x +41.506). The dataset indicates no significant changes in human diets across the three time periods represented.

Stable isotope values are available for eight Priol’khon’e canids, including three Early Neolithic dogs, two Early Bronze Age dogs, one Late Bronze Age dog, and one probable wolf and one dog from the Iron Age (Figure 14). The Neolithic and Early Bronze Age dogs, all presumably living with foragers, have only a slightly higher average δ^15^N value (mean = 13.2±0.4‰) than that of the two later dating dogs (mean = 12.4±0.5‰), suggesting these later dating pastoralists’ dogs relied marginally less on aquatic foods than the older dating dogs. The range in δ^13^C values for all dogs (−18.6‰ to –17.0‰) overlaps well with upper portion of the range of human δ^13^C values; these humans appear to have had the greatest reliance on aquatic foods within the Priol’khon’e population. The dogs also tend to have lower δ^15^N values than humans with similar δ^13^C values, similar to the pattern seen among some of the Angara River dogs; these dogs appear to have had diets rich in protein from aquatic foods. The Khotoruk specimen’s isotope values mark it as a clear outlier, and indicate that it made little use of aquatic foods, consistent with its genetic identification as a wolf.

## Discussion

The earliest dog potentially evidenced in Cis-Baikal’s archaeological record is represented by scattered skeletal remains at Ust’-Khaita, and dates to the Late Pleistocene, a period in which human subsistence practices appear to have been focused on large ungulates, including deer. The first canid burial, which dates to just over 8000 years ago, is the wolf interred with human remains at the Lokomotiv cemetery. Dog burials, some with human skeletons and some in their own burial pits, appear several centuries later in both study subareas (but not the Upper Lena) within the Early Neolithic period, at sites Shamanskii Mys, Shamanka II, and Pad’ Kalashnikova. This same period is characterized by the widespread appearance of human graves and cemeteries across Cis-Baikal, and other ritualized treatments of animal remains (Table 1).

Human diets in the Angara/South Baikal region during the Early Neolithic were variable, but generally included some aquatic foods, and faunal assemblages from this subregion and time period are dominated by deer and fish [10], [51]. The buried dogs found there, all dating to the Early Neolithic period, had variable diets, with some relying heavily on aquatic foods, others much less so. Human diets in Priol’khon’e during the Middle Holocene were variable, primarily in regard to individuals’ reliance on local aquatic foods [14], [15]. Forager habitation sites on the Little Sea coast of Priol’khon’e are dominated by littoral fishes, with terrestrial mammals and seals being present in trace amounts only [16], [52]. Site faunal assemblages on the open coast of Priol’khon’e from any part of the Holocene are dominated by seal remains, but domesticated ungulates are common in deposits post-dating ∼3000 B.P [9], [53]. Small quantities of deer remains are present in these sites, particularly on the open coast. All Middle Holocene dogs analyzed from this subregion, including buried and non-buried individuals, had diets with substantial aquatic content, with a mean δ^13^C value similar to that of the Priol’khon’e foragers with aquatic-rich diets, and a mean δ^15^N value slightly below this group’s average–they were eating much like the local humans who were most reliant on aquatic foods.

Dog burials appear to have been absent in the third Cis-Baikal subregion, the Upper Lena, and Middle Holocene foragers buried there had the lowest mean δ^15^N values across all of Cis-Baikal (mean = 11.0‰; [15]), suggesting a greater reliance on terrestrial mammals than in the other two study subregions. Further, there also is no evidence of dog burials during the Early Holocene, when subsistence adaptations seem to be focused on ungulates. The advent and continued practice of foragers burying dogs in Cis-Baikal then is clearly temporally correlated with the practice of regularly burying humans (and sometimes other animals) in this region, but not with a single dietary pattern, and certainly not with a human dietary focus on terrestrial mammals. The opposite is true–dogs were most commonly buried in areas where foragers tended to have diets rich in aquatic foods. In Priol’khon’e and South Baikal, these aquatic foods likely included littoral fishes and Baikal seals, the latter of which dogs could have been used to help procure. On the Angara, however, aquatic fauna are largely limited to riverine fishes, and dogs likely were of little use in obtaining these. Clearly, no single human subsistence practice or diet is correlated with the practice of burying dogs in Cis-Baikal. If anything, dogs were more commonly buried where diets were broader as a result of use of both terrestrial and aquatic fauna.

Offsets between the δ^13^C and δ^15^N values of dogs and humans have been noted in previous studies, with the most common pattern being one where dogs show lower δ^15^N values, or both lower δ^13^C and lower δ^15^N (see Table S1). In the Angara River dataset, dog δ^15^N values are offset from those of humans only when both species are eating diets rich in aquatic foods (Figure 13). In Priol’khon’e, all dogs had diets rich in protein from aquatic sources, and on average have lower δ^15^N values than humans with similar δ^13^C values (Figure 14). The most commonly suggested cause of lower δ^15^N values in dogs than in humans is that dogs were consuming human feces, which was thought to be depleted in ^15^N compared to the human diet (see references in Table S1). However, recent studies of δ^13^C and δ^15^N values in the feces of humans fed controlled diets found no significant difference in the stable isotope ratios of the feces and the diets [54]. This suggests that dogs eating feces is unlikely to account for the δ^15^N offsets seen in our data. Another proposed explanation for isotopic offsets between canids and humans has been bone collagen ingestion [55]. In mammals, the δ^15^N value of bone collagen is essentially the same as the muscle, while the δ^13^C value of bone collagen is enriched by ∼4‰ relative to the muscle [56]. This is a poor fit to our data because dogs consuming more collagen from mammal bones than humans would show higher δ^13^C, not lower δ^15^N, and the offset should also have affected dogs eating diets with little aquatic protein content; such a pattern clearly is not evident in our samples. Less is known about the isotopic values of fish bone relative to muscle. At least one study indicates that fish bone has a lower δ^15^N value than fish muscle while another suggests it is higher [57], [58]. An alternative explanation for the offsets in our samples may be that when fish were eaten, humans preferentially kept and consumed larger fish, which would have higher δ^15^N values, and gave the smaller fish to their dogs. Although humans and canids likely shared many foods, their diets would not have been identical in almost any circumstance. Humans may have retained some foods for themselves and canid diets presumably included a number of items such as mammal bones, fish bones and viscera that humans consumed less frequently, potentially leading to isotopic differences.

While some Lake Baikal fauna consumed by humans clearly carry an old carbon offset when radiocarbon dated [9], what factors contribute to this offset, its potential variability within the lake fauna, and its magnitude in fish in the lake’s tributaries remain unknown. Regardless, the degree to which humans and dogs relied on local aquatic foods likely will correlate to some degree with the magnitude of offset in their radiocarbon ages. The general similarity in Cis-Baikal dog and human diets suggests both would carry an old carbon bias when radiocarbon dated, with dietary differences between the two species potentially producing different magnitudes of age offset. The Lokomotiv wolf grave provides a useful example of maximum magnitude of offset to expect between humans and canids. Humans from Lokomotiv had diets rich aquatic-foods (δ^13^C mean = −15.75±0.8‰; δ^15^N mean = 14.15±0.7‰), while the wolf was eating very little of these foods (δ^13^C = −20.2‰; δ^15^N = 9.7‰). Correspondingly, the two radiocarbon dates on human remains buried with the wolf were ∼500 radiocarbon years older than the dates on the canid’s skeleton [2]; this magnitude of offset might characterize most of the radiocarbon dates on human remains in this cemetery. The Angara River dogs in this study, all but one of which date to the Early or Middle Neolithic (the Ust’-Khaita canid, excluded here), also have lower mean δ^15^N and δ^13^C values (δ^13^C mean = −16.8±1.5‰; δ^15^N = 11.3±0.6‰) than the Angara Early Neolithic human group (δ^13^C mean = −15.8±0.8‰; δ^15^N average = 14.2±0.7‰), but these dietary differences are of far lower magnitude than that between the Lokomotiv wolf and humans (Figure 13). This suggests that the Angara River dogs’ age offsets are of lower magnitude than that inferred for the human remains for this region. In Priol’khon’e, dog and human diets also are relatively similar. However, the dogs here appear to have eaten at slightly higher trophic levels than at least some locally-buried humans, and at lower levels than many others, suggesting there is a more complex pattern in the errors to be expected from the dates on human and canid remains in this region. Regardless, we believe the data suggest the chronology for the Cis-Baikal dogs is not biased to the extent that the overall temporal patterns in dog burial practices cannot still be inferred with reasonable confidence. Precise corrections to Cis-Baikal’s radiocarbon dates is probably only possible through future paired dating of directly associated remains of terrestrial herbivores and dog or human skeletons.

Proceeding with the assumption that the canid radiocarbon dates are providing a reasonably reliable indication of their place in the culture history model, additional temporal patterns can be examined. No dog remains examined in this study date to the Late Neolithic period, and even if all dog radiocarbon dates were decreased in age by 3–4 centuries, only a single specimen, Ust’-Belaia dog #3, would fall within this period as it is currently defined, and then only marginally so. Unadjusted, this specimen dates firmly within the Middle Neolithic period, when human burials are very rare; this specimen also is not from a formal burial. It is possible that dogs were rare during this period in general, or perhaps the pattern is a result of curation and sampling issues. Within Cis-Baikal, the Angara River area appears to have contained the greatest number of Late Neolithic sites, but most of these were excavated in the 1970s or earlier and the collections are now lost [59]. Human burials have been dated to this period, but are notably far fewer in number than those from the Early Neolithic or Early Bronze Age [3]. Perhaps the lack of Late Neolithic dogs relates to the overall dearth of Late Neolithic archaeological materials now available.

We also have no primary dog burials dating to the Early Bronze Age, although a possible secondary dog burial was present within a human grave at Uliarba II, and other dog remains in the region clearly date to this period. Further, an Early Bronze Age primary dog burial at the Pad’ Lenkovka cemetery on the Angara River previously has been reported [60] (this specimen could not be relocated for study), suggesting that at least some dogs were buried during this period. Overall, dog burials were most common during the Early Neolithic, absent in the Middle Neolithic, and rare in the Late Neolithic and Early Bronze Age. This trend occurs on the Angara, where human diets had apparently shifted to less reliance on fish by the start of the Late Neolithic, and is observed in Priol’khon’e, where there is no evidence for a change in human dietary patterns across the entire Middle Holocene.

The Late Holocene in Cis-Baikal, which is marked in part by the presence of pastoral groups and various human mortuary traditions, also lacks clearly identifiable dog burials in human cemeteries. The possible exception is the Todakta I dog, which we have interpreted as part of the sacrifice of several domesticated animals associated with human burials at this site. Pastoralist groups by definition lived in close association with many domesticated species, here most often sheep, goats, horses, and cattle. These groups regularly rode, sold, traded, sacrificed, and consumed these animals, and considered them property. Perhaps because dogs in these societies no longer had the unique position of being humans’ only cohabitant animal, and because people’s relationships to animals more broadly had changed with the emergence of pastoralism, dogs no longer were considered to have spiritual equivalency with humans and were no longer considered eligible for burial in human cemeteries.

The mtDNA recovered from five of the Cis-Baikal canids provides a first picture of the genetics of ancient dogs from East Asia, and also another wolf. The dog specimens yielding mtDNA all belonged to dog clade I, which also includes Middle and Late Holocene archaeological dogs from Europe, West Asia, Alaska, and Central and South America [33], [47], [61] and modern dogs on several continents [45], [46]. Our samples show this clade was present in East Asia since at least the Middle Holocene. Notably, most Pre-Columbian American dogs also belong to clade I [33], [62], [63]. Further, the mtDNA haplogroup identified in an Early Neolithic dog from Pad’ Kalashnikova was also found in an Early Bronze Age dog from Ulan-Khada. This may suggest at least some continuity in dog populations here during the Middle Holocene, a period in which discontinuity in human haplogroups has been documented [6], [7], although this haplotype is currently widespread and so could have been common and/or widespread in the past as well.

The wolf haplotypes identified in our samples do not appear to have contributed genetically to the local dog population, but additional DNA analyses of ancient wolves in the area are needed to further verify this. The haplotype in the Khotoruk wolf matches a haplotype found in living Russian wolves, indicating some level of population continuity for the last 1400 years or so.

### Conclusion

Dogs appear to have lived among humans in Cis-Baikal throughout much of the Holocene, and perhaps since the very Late Pleistocene. Middle Holocene foraging groups here seem to have been the only societies that provided dogs formal burials within cemeteries otherwise used for human interment. In other words, these groups treated select dogs much like humans upon their deaths, interring some in their own graves with various accoutrements, and others in graves also bearing humans. This occurred where human and dog diets were relatively broad and included significant aquatic components, and not where and when they were primarily focused on ungulates. Dogs would seemingly have been useful in hunting these terrestrial mammals, but there is no evidence for them being buried in any period on the Upper Lena, nor are there any documented dog burials during the Early or Late Holocene, the former characterized by a human focus on hunting ungulates, the latter by pastoralism, ungulate hunting, and fishing. Even where and when dogs were buried by foragers, simple relationships between human dietary patterns and dog burials is not evidenced, as dogs were buried where groups were particularly reliant on riverine fish and ungulates, and also among groups who were hunting seals, fishing in Lake Baikal, and taking some deer. Human emotional attachment to dogs alone cannot account for these patterns, as both humans and dogs almost certainly would have had the capacity for such close personal relationships across all of Cis-Baikal and throughout the last 12,000 years. The evidence presented here indicated that the foragers in Cis-Baikal burying dogs in cemeteries did this only when they were regularly burying their human dead (the Middle Holocene) in such settings. Dogs were the only domesticated animals living with humans at this time, and it appears that dogs and animals such as bears [64] were considered by foraging groups here to be spiritually similar to humans, as were many animals among historic northern indigenous groups [65]. When these broader beliefs were combined with intimate personal relationships with dogs, which here involved sharing many of the same foods, and the broader practice of burying one’s group members in cemeteries, some dogs were given ‘human’ mortuary rites.

How the Cis-Baikal dogs were being used by their human counterparts remains unclear, but it is apparent from the way that at least some of these animals were treated at death that they considered more than mere bio-technologies–they were parts of people’s emotional and social lives. Dogs in such settings likely held a number of roles, even within their individual lifetimes, ranging from companionship or guardian to fellow hunter and burden carrier. Further details of these roles can only emerge when the life histories of ancient dogs are explored through detailed studies of their skeletons’ chemistry and morphology, and with the integration of this data with archaeological contextual information. Stable isotope studies of dog diets are one step in this process, and help move the study of domestication solely from a search for deep origins to an exploration of human-animal interaction.

## Supporting Information

Table S1 1a. Comparison of human and dog collagen stable carbon and nitrogen isotope values from other studies. Criterion for inclusion in the table is that the data represent multiple dogs and humans clearly from the same site and time period. Table S1b. Studies excluded in table S1a but which were reviewed because they have multiple isotope values for both dogs and humans.
(DOCX)
