# Supplementary material for: Burying Dogs in Ancient Cis-Baikal, Siberia: Temporal Trends and Relationships with Human Diet and Subsistence Practices
Source: PLoS One. 2013 May 17;8(5):e63740. doi: 10.1371/journal.pone.0063740 (PMC3656967; doi:10.1371/journal.pone.0063740)
Supplement: Table S1 — 1a. Comparison of human and dog collagen stable carbon and nitrogen isotope values from other studies. Criterion for inclusion in the table is that the data represent multiple dogs and humans clearly from the same site and time period. Table S1b. Studies excluded in table S1a but which were reviewed because they have multiple isotope values for both dogs and humans. (DOCX) [file pone.0063740.s001.docx]

Table S1a. Comparison of human and dog collagen stable carbon and nitrogen isotope values from other studies. Criterion for inclusion in the table is that the data represent multiple dogs and humans clearly from the same site and time period.

| **Source** | **Site/region** | **# human/dog** | **Date (years BP)** | **Δ^13^C_(human-dog)_** | **Δ^15^N_(human-dog)_** | **Major human diet components** |
| --- | --- | --- | --- | --- | --- | --- |
| [1]^1^ | Aitutaki, Cook Islands | 8/5 | c. 700–500 | -2.5 | -0.6 | Marine; terrestrial |
| [2]^2^ | Vlasac, Serbia | 36/10 | c. 11,000–8,500 | 0.3 | 3.5 | Freshwater; terrestrial |
| [3] | Namu, Canada | 13/13 | c. 6,000–2,000 | -0.1 | 2.6 | Marine/anadromous; terrestrial |
| [4]^3^ | Kopingsvik, Sweden | 18/11 | c. 5,600–3,800 | 0.4 | 1.9 | Marine mammals and fish |
| [5]^4^ | Korsnäs, Sweden | 8/4 | c. 5,400–4,600 | -0.5 | 1.8 | Marine mammals |
| [6]^5^ | Montou, France | 5/4 | c. 6,000–5,500 | 0.0 | -0.2 | Terrestrial (domestic and wild) |
| [7] | Santa Rosa Island, USA | 15/5 | c. 1,300–200 | -2.0 | -0.5 | Marine; terrestrial |
| [8] | Cuello, Belize | 28(23)^6^/12 | c. 3,200–1,600 | 2.7 | 1.4 | Maize; terrestrial; freshwater |

1. Period I samples only.

2. Humans exclude neonates and infants.

3. Humans exclude juveniles, and are for Mesolithic and Neolithic only.

4. Humans exclude juveniles.

5. Humans exclude ‘child 3’ juvenile.

6. Human n 28 for δ^13^C, 23 for δ^15^N.

Table S1b. Studies excluded in table S1a but which were reviewed because they have multiple isotope values for both dogs and humans.

| **Source** | **Site/region** | **Reasons for exclusion and notes on patterning** |
| --- | --- | --- |
| [9] | Northeastern United States | The same-site data set is limited to one dog and three humans, with no δ^15^N values available for the human samples. Patterning: dog δ^13^C is slightly lower than human δ^13^C |
| [10] | Denmark | The data sample many sites varying in locale and date and include no clearly contemporaneous same-site sets of multiple dogs and humans. Patterning: the aggregate data show a pattern similar to this study, with dogs lying along a shallower δ^13^C- δ^15^N slope than humans (see pg. 2144). |
| [4] | Resmo and Torsborg, Sweden | The Resmo dogs are not dated, making comparability unclear as human diets at Resmo varied over time. At Torsborg only two dogs were analysed, and one of these is outside the date range of the humans. Patterning: at Resmo dogs largely overlap the human range for δ^15^N and δ^13^C but show two outlying δ^13^C values well above the highest human δ^13^C value. At Torsborg dogs fall within the human δ^15^N and δ^13^C ranges. |
| [11] | Eastern United States | The same-site data set is limited to one dog and two humans (two additional dogs from a nearby site are also analysed). Patterning: dog δ^13^C values overlap the human δ^13^C range but are somewhat higher; dog δ^15^N values are somewhat below human δ^15^N values (no overlap). |
| [12] | Ontario, Canada | The human and dog samples are not from the same site, although the sites are nearby (48 km. apart) and roughly contemporaneous. Patterning: dog δ^13^C values overlap the human δ^13^C range but are somewhat higher; dog δ^15^N values are well below human δ^15^N values (no overlap). |
| [13] | Hanamiai, French Polynesia | The humans samples are not dated, making comparability unclear as trends in the dog remains show that marine use changed over time. Patterning: dog δ^13^C values overlap the human δ^13^C range but are somewhat higher; dog δ^15^N values are well below human δ^15^N values (no overlap). |
| [14] | Ohio Valley, United States | The same-site data set includes only one dog and five humans. Patterning: dogs are within the human range for both δ^15^N and δ^13^C. |
| [14] | Nicaragua (modern) | Directly comparable human and dog samples consist of hair (as opposed to the collagen values given in table S1). Patterning: human and dog hair from the same household are similar, with dogs showing slightly elevated δ^15^N relative to humans and similar δ^13^C. |

**References Cited**

1. Allen MS, Craig JA (2009) Dynamics of Polynesian subsistence: insights from archaeofauna and stable isotope studies, Aitutaki, Southern Cook Islands. Pac Sci 63:477-506.

2. Boric D, Grupe G, Peters J, Mikic Z (2004) Is the Mesolithic-Neolithic subsistence dichotomy real? New stable isotope evidence from the Danube Gorges. Eur J Archaeol 7:221-248.

3. Cannon A, Schwarcz HP, Knyf H (1999) Marine-based subsistence trends and the stable isotope analysis of dog bones from Namu, British Columbia. J Archaeol Sci 26:399–407.

4. Eriksson G, Linderholm A, Fornander E, Kanstrup M, Schoultz P, et al. (2008) Same island, different diet: Cultural evolution of food practice on Öland, Sweden, from the Mesolithic to the Roman Period. J Anthropol Archaeol 27:520–543.

5. Fornander E, Eriksson G, Lidén K (2008) Wild at heart: Approaching Pitted Ware identity, economy and cosmology through stable isotopes in skeletal material from the Neolithic site Korsnäs in Eastern Central Sweden. J Anthropol Archaeol 27:281-297.

6. Le Bras-Goude G, Claustre F (2009) Exploitation of domestic mammals in the Eastern Pyrenees during the Neolithic: human dietary patterns at the site of Montou (Corberes-Les-Cabanes, France) using bone collagen stable isotopes (δ^13^C, δ^15^N). Vie Milieu 59:215-221.

7. Rick TC, Culleton BJ, Smith CB, Johnson JR, Kennet DJ (2011) Stable isotope analysis of dog, fox and human diets at a Late Holocene Chumash village (CA-SRI-2) on Santa Rosa Island, California. J Archaeol Sci 38:1385-1393.

8. Tykot RH, Falabella F, Planella MT, Aspillaga E, Sanhueza L, Becker C (2009) Stable isotopes and archaeology in Central Chile: methodological insights and interpretive problems for dietary reconstruction. Int J Osteoarchaeol 19:156-170.

9. Allitt S, Stewart RM, Messner T (2008) The utility of dog bone (Canis familiaris) in stable isotope studies for investigating the presence of prehistoric maize (Zea mays ssp. mays): a preliminary study. N Am Archaeol 29:343-367.

10. Fischer A, Olsen J, Richards M, Heinemeier J, Sveinbjörnsdóttir ÁE, Bennike P (2007) Coast–inland mobility and diet in the Danish Mesolithic and Neolithic: evidence from stable isotope values of humans and dogs. J Archaeol Sci 34:2125–2150.

11. Hogue SH (2003) Corn dogs and hush puppies: diet and domestication at two protohistoric farmsteads in Oktibbeha County, Mississippi. Southeast Archaeol 22:185-195.

12. Katzenberg MA (1989) Stable isotope analysis of archaeological faunal remains from Southern Ontario. J Archaeol Sci 16:319-329.

13. Richards MP, West E, Rollet B, Dobney K (2009) Isotope analysis of human and animal diets from the Hanamiai archaeological site (French Polynesia). Archaeol Ocean 44:29-37.

14. Takersley KB, Koster JM (2009) Sources of stable isotope variation in archaeological dog remains. N Am Archaeol 30:361-375.
